# The persistence of smoke VOCs indoors: Partitioning, surface cleaning, and air cleaning in a smoke-contaminated house

**DOI:** 10.1126/sciadv.adh8263

**Published:** 2023-10-13

**Authors:** Jienan Li, Michael F. Link, Shubhrangshu Pandit, Marc H. Webb, Kathryn J. Mayer, Lauren A. Garofalo, Katelyn L. Rediger, Dustin G. Poppendieck, Stephen M. Zimmerman, Marina E. Vance, Vicki H. Grassian, Glenn C. Morrison, Barbara J. Turpin, Delphine K. Farmer

**Affiliations:** ^1^ Department of Chemistry, Colorado State University, Fort Collins, CO 80523, USA.; ^2^ National Institute of Standards and Technology, Gaithersburg, MD 20899, USA.; ^3^ Department of Chemistry and Biochemistry, University of California San Diego, La Jolla, CA 92093, USA.; ^4^ Department of Environmental Sciences and Engineering, Gillings School of Global Public Health, The University of North Carolina at Chapel Hill, Chapel Hill, NC 27599, USA.; ^5^ Department of Mechanical Engineering, University of Colorado Boulder, Boulder, CO 80309, USA.

## Abstract

Wildfires are increasing in frequency, raising concerns that smoke can permeate indoor environments and expose people to chemical air contaminants. To study smoke transformations in indoor environments and evaluate mitigation strategies, we added smoke to a test house. Many volatile organic compounds (VOCs) persisted days following the smoke injection, providing a longer-term exposure pathway for humans. Two time scales control smoke VOC partitioning: a faster one (1.0 to 5.2 hours) that describes the time to reach equilibrium between adsorption and desorption processes and a slower one (4.8 to 21.2 hours) that describes the time for indoor ventilation to overtake adsorption-desorption equilibria in controlling the air concentration. These rates imply that vapor pressure controls partitioning behavior and that house ventilation plays a minor role in removing smoke VOCs. However, surface cleaning activities (vacuuming, mopping, and dusting) physically removed surface reservoirs and thus reduced indoor smoke VOC concentrations more effectively than portable air cleaners and more persistently than window opening.

## Teaser

Wildfire smoke persists indoors due to equilibrium partitioning to surfaces, but surface cleaning is effective to reduce exposure.

## INTRODUCTION

Wildfires are increasing in frequency and severity due to human behavior, climate change, and population growth at the wildland-urban interface (*1*, *2*). This growing incidence of wildfires has created an emerging public health challenge because wildfire smoke contains many primary and secondary air pollutants of concern (*3*–*5*). Smoke-associated pollutants include particulate matter (PM), carbon monoxide (CO), volatile organic compounds (VOCs), polycyclic aromatic hydrocarbons, and ozone (*6*–*9*). Wildfire smoke can infiltrate indoors (*10*–*12*), which modifies exposure and raises the need for evidence-based approaches to create cleaner indoor air spaces where residents can seek refuge (*13*–*15*).

The chemistry of indoor building environments is distinct from that of the outdoor atmosphere, partly due to high surface area–to–volume ratios (*15*, *16*). The vast indoor surface area hosts chemicals that partition from the air to the surface, generating reservoirs that can hold far greater levels of contaminants than air (*15*, *17*). As smoke transports indoors, surface reservoirs drive partitioning processes and, as is the case for third-hand cigarette smoke, reactive chemistry (*17*–*21*). Here, we consider surface reservoirs as the condensed phase compartments that interact with the gas phase over time scales of seconds to years, including both the air-surface interface and the deeper bulk reservoir (*16*). These surface reservoirs are complex and likely spatially heterogeneous (*22*–*24*), although few studies have been able to directly probe their composition and behavior (*25*–*29*). Previous chamber and field studies have illustrated the partitioning of gas-phase molecules to and from various indoor surfaces such as flooring, carpet, painted wall board, ceiling tile, and window glass (*21*, *26*, *30*–*33*). For example, 3-ethenylpyridine (3-EP) and nicotine were not observed in the gas phase 2 hours after the cessation of cigarette smoking, likely due to quick absorption onto surfaces, whereas other VOCs remained present in the gas phase for at least 18 hours (*21*). Indoor VOC concentrations respond to these adsorption and desorption processes and can be used to determine corresponding rate coefficients and equilibrium partitioning (*34*–*36*). Together, these studies suggest that the complex mixture of wildfire smoke could partition to indoor surfaces and create indoor pollutant reservoirs from which gases reemit. However, the extent to which smoke VOCs will persist in surface reservoirs (versus remain in air and be removed by ventilation) will control the time scale of subsequent release of those VOCs and thus potential indoor smoke VOC exposure. The molecular properties that control this partitioning and the associated time scales for interactions between smoke VOCs and indoor residential surfaces are unclear.

Individuals may have limited capacity to control outdoor air pollution entering their homes, but established strategies to improve indoor air quality include reducing the sources of VOCs, increasing ventilation of clean air, and using air cleaners (*14*). However, surface reservoirs may provide a hidden, persistent source of VOCs to indoor air. Studies conducted in residential buildings show that most of the volatile organic and inorganic contaminant mass resides in surface reservoirs, rather than air (*26*, *37*). When ventilation is stopped, indoor VOC concentrations return to preventilation levels within a few hours due to emissions from surface reservoirs (*19*). These observations raise questions about the long-term benefits of air cleaning approaches; while often effective at reducing PM levels, air cleaners may only have temporary ability to reduce airborne VOC concentrations (*38*). Surface cleaning methods such as mopping, dusting, and vacuuming have the potential to directly reduce indoor surface reservoirs, but this concept remains unexplored in real-world environments. Indoor surface cleaning studies typically focus on the potential for detergent solutions to emit into the gas phase or induce chemical reactions; these emissions can contribute VOCs to indoor air, but the emissions are typically temporary (hours) in nature (*39*, *40*).

To probe the capacity for smoke emissions to contribute to indoor surface reservoirs, we added smoke from burned pinewood chips into a well-characterized test house (*41*, *42*) and measured the consequent changes in chemical composition of the indoor environment. We used the observed concentration decays of smoke VOCs to characterize adsorption and desorption processes and develop a model describing indoor partitioning. This model allowed us to predict time scales for persistence of smoke VOCs in indoor environments and estimate the three fates for VOCs in the house: continued existence in the gas phase, contribution to surface reservoirs, or removal by ventilation. To investigate the efficiency of different cleaning strategies on indoor surface and air reservoirs, we conducted a post-smoke cleaning study using different air and surface cleaning activities, including running a portable air cleaner, surface cleaning (dusting, vacuuming, and mopping), and extensive ventilation with all the doors and windows open. These tests quantify the remarkable capacity of indoor surfaces to store VOCs and demonstrate the need to consider gas-surface partitioning when designing and implementing air cleaning methods for indoor VOC removal. Although this paper focuses on wildfire smoke, our results may apply to other air pollution scenarios, including intensive cooking, infiltration of heavy urban smog, cigarette smoking, or other indoor emission activities.

## RESULTS

### Dynamic gas-surface partitioning drives indoor gas concentrations

**Fig. 1.**
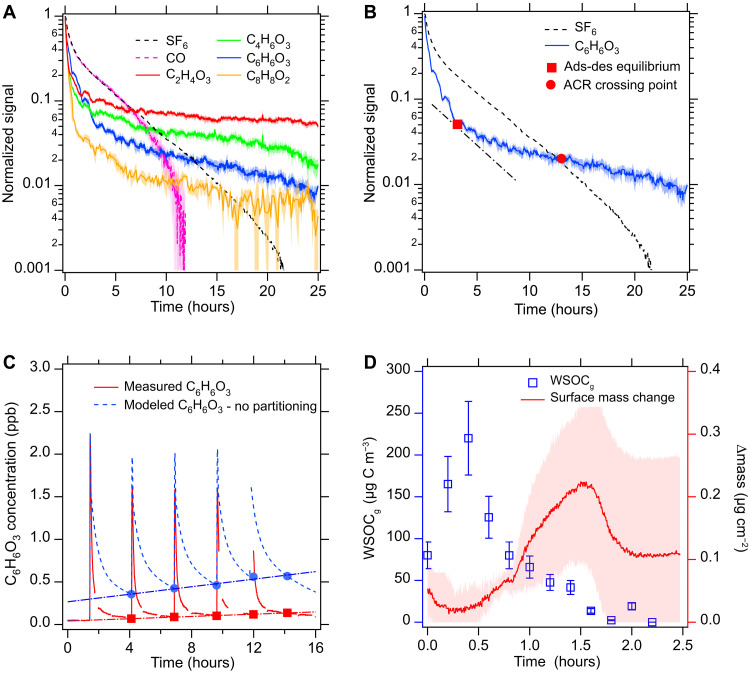
Gas-phase compound decay patterns and gas-surface partitioning dynamics during smoke events. (**A**) Normalized signal decay of four gas-phase compounds after smoke addition in the house. SF_6_ is an inert tracer used to estimate the ACR. CO is an inert smoke tracer with an initial decay that is consistent with SF_6_; after 8 hours, CO levels near background conditions and deviations are difficult to distinguish from noise. Shading represents the SD of 10-s data when averaged to 2-min intervals. (**B**) Adsorption-desorption equilibrium point (ADP) and ACR crossing point (ACP) are shown for exemplary C_6_H_6_O_3_. The dashed-dotted line is a tangent line defining the ADP point. (**C**) Repeated smoke additions show decay patterns consistent with partitioning theory for C_6_H_6_O_3_. Points (red squares and blue dots) are reference points used to describe the difference between the measurement and model cases. (**D**) Concentration of total gas-phase water-soluble organic carbon (WSOC_g_; blue squares) and surface mass (red line) vary with time after smoke injection due to surface partitioning. Error bars show measurement uncertainty. The red line is the average mass change of three smoke additions in fig. S9, and shading shows SD of additions. ppb, parts per billion.

High levels of outdoor air pollution during smoke events can enable substantial pollutant infiltration to indoor spaces (*10*), followed by gas-surface partitioning (*18*). Figure 1A shows the normalized concentration decay of four example compounds after adding wood smoke to the house, including peracetic acid (C_2_H_4_O_3_), acetoacetic acid (C_4_H_6_O_3_), hydroxymethylfurfural (C_6_H_6_O_3_), and *o*-toluic acid (C_8_H_8_O_2_) (*7*, *8*). Gas-phase organic molecules with higher molecular weights tend to decay faster, indicating faster surface adsorption. SF_6_ and CO tracers provide air change rate (ACR) and enable correction for the dilution effect of indoor air mixing (section S2). Assuming a well-mixed gas phase and mass balance, the rates of change in concentration of compound *i* in the gas phase (*G_i_*) and indoor surface (*W_i_*) can be expressed as:

$$\frac{d[G_{i}]}{dt}=-\lambda [G_{i}]-\lambda_{a}[G_{i}]+\lambda_{d}[W_{i}]$$ (1)

$$\frac{d[W_{i}]}{dt}=\lambda_{a}[G_{i}]-\lambda_{d}[W_{i}]$$ (2)

where *G_i_* and *W_i_* are the total mass in each sink normalized to room air volume (μg m^−3^), λ is the ACR (hour^−1^), and λ_a_ and λ_d_ are first-order rate coefficients of surface adsorption and desorption (hour^−1^). Here, we describe the first-order dynamic mass distributions between two sinks in a typical ventilation condition and the related implications to indoor air quality. In Fig. 1B, we propose two time scales to describe indoor partitioning: the adsorption-desorption equilibrium point (ADP; red square) and the ACR crossing point (ACP; red dot). The ADP represents the time point at which gas-surface partitioning reaches a temporary equilibrium state when gas–to–surface mass transfer λ_a_[*G_i_*] is equal to surface–to–gas mass transfer λ_d_[*W_i_*]. C_6_H_6_O_3_ initially decays faster than SF_6_ before its ADP (3.1 hours) due to rapid surface adsorption, and then slower after the ADP due to source contribution from surface desorption. ADP is observationally derived as the time at which the tangent to the concentration decay curve of the gas (i.e., C_6_H_6_O_3_) is parallel to the linear (or post-dilution) section of SF_6_ decay, reflecting the moment at which the first-order rate constant is equal to the indoor ACR, and there is neither net surface adsorption nor desorption, although this equilibrium is temporary. Indoor environments are inherently dynamic due to the continuous exchange of mass and energy with surrounding environment (*43*). After reaching ADP, the mass reductions for the gas and surface mass are mainly driven by the ventilation term λ[*G_i_*] and desorption term λ_d_[*W_i_*], respectively. We define ACP as the time at which the concentration decay curves of the smoke VOC and SF_6_ intersect, implying that the role of indoor surfaces has changed at this moment. Before ACP, the net effect of gas-surface partitioning is to reduce smoke VOC concentrations more rapidly than SF_6_ and thus clean room air; after ACP, the surface reservoirs can serve as gas emission sources for hours, days, and perhaps even years (*17*). Figure S5 shows the extended persistence of C_6_H_6_O_3_ in room air for 78 hours, implying a continuous surface emission with a time scale that varies as the air is ventilated and the surface-gas equilibrium constantly shifts. The characteristic time scale (τ) for C_6_H_6_O_3_ persistence increased from 0.9 hours on day 1 to 9.7 hours on day 2 to 25.1 hours on day 3, corresponding to first-order decay rates (1/τ) of 1, 0.1, and 0.04 hours^−1^, respectively.

Residential exposure to outdoor woodsmoke will depend on the nature of the fire (proximity, severity, and fuel), meteorological conditions (wind direction and force scales), and building-specific infiltration factors (*10*, *14*). Infiltration of air pollution can thus be a continuous process for an extended time period (e.g., presence of regional wildfires over days or weeks) or a sporadic process on short time scales (e.g., residential wood burning or brief exposure as a wildfire is suppressed). The red curves in Fig. 1C show replicable decay patterns—and increasing backgrounds—of C_6_H_6_O_3_ concentrations during repeated smoke addition events. The average aerosol mass concentration during each injection is around three times that of the average smoke exposure experienced in California homes on wildfire-influenced days (mean indoor PM_2.5_ of 11.1 ± 8.3 μg m^−3^) (*10*). A more comprehensive examination of chemical compositions of submicron aerosols and VOCs can be found in figs. S6 and S7, respectively. As our smoke injections were short in duration (minutes), the test house’s total smoke exposure over the course of a day was comparable to real-world conditions. This replicable decay suggests that indoor surface reservoirs have such great adsorption capacity even after extended pollution exposure that most VOC concentrations may drop quickly once the outdoor infiltration stops. To further explore this concept, the blue dashed curves represent C_6_H_6_O_3_ concentration models, assuming no surface partitioning occurs, only dilution and ventilation. To quantify the impact of including surface partitioning on C_6_H_6_O_3_ concentrations, we choose reference points ~2.5 hours after each smoke injection for the no-partitioning (blue square) versus with-partitioning (red square) cases. The post-smoke injection concentration for the no-partitioning case is 4.1 to 5.2 times higher than that including surface partitioning, with fitted slopes of 0.022 and 0.007 parts per billion/hour, respectively. This trend demonstrates that partitioning to surface reservoirs substantially lowers indoor VOC air concentrations during outdoor smoke and wildfire events. Excluding this surface partitioning effect and only considering infiltration, ventilation, and dilution would overestimate the acute exposure of VOCs. In general, the indoor VOC concentration is initially (i.e., before ACP) driven by the injected or infiltrated concentrations and dilution; on longer time scales, VOC concentrations are driven by the surface-accumulated mass. Further, in contrast to PM, which can be effectively removed through filters in the heating, ventilation, and air conditioning (HVAC) systems, mechanical or natural ventilation during wildfire events will increase the VOC surface accumulation and thus subsequent exposure after polluted days.

Surface reservoirs play an important role in indoor air quality but can change rapidly in response to indoor environmental conditions. Figure 1D shows the concentration decay of total gas-phase water-soluble organic carbon (WSOC_g_) after a smoke addition event (in blue) and the subsequent surface adsorption and desorption illustrated as the measured mass change of the deposited surface organic film (in red) by a thin porous TiO_2_ film deposited on a quartz crystal microbalance (QCM). Around 2 hours after adding smoke, the concentration of WSOC_g_ decreased by >90%, consistent with the fast surface adsorption observed for C_6_H_6_O_3_ (Fig. 1B). The highest point of the surface mass potentially corresponds to the ADP of total gas-phase masses when the net gas-surface mass transfer is zero. A mathematical derivation for this correspondence is presented in the section S5. While the mass change of each cycle varied in magnitude, the shapes were consistent (fig. S9). This consistent shape was a most notable feature of the partitioning curves for the indoor surface films, implying highly dynamic and responsive gas-surface partitioning. Figure S9A highlights the tendency of the smoke-exposed, dirty TiO_2_ thin film to lose its surface reservoir capacity gradually with repeated smoke additions. This exposure-driven loss in surface capacity implies that the film of materials deposited on indoor surfaces has different partitioning properties toward VOCs than the underlying crystal substrate. Thicker smoke films may cause longer ADP time scales, as observed for smoke additions of 0.35 and 0.5 g of woodchips (fig. S9B). This surface chemical evolution is likely influenced by not only indoor VOC concentrations but also relative humidity (RH) (*44*, *45*), indoor air flow, particle deposition, and temperature (*46*, *47*). However, it is the capacity of the reservoir that enables the observed persistence of smoke VOC emissions and elevated air concentrations.

### Volatility affects partitioning behavior and fate of VOCs

**Fig. 2.**
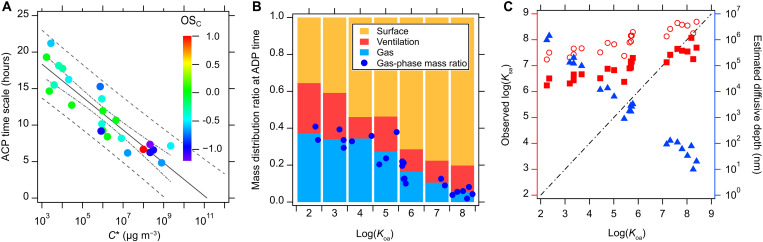
Volatility characteristics affect VOC partitioning behaviors. (**A**) ACPs of smoke VOCs correlate with saturation concentration (C*) on a logarithmic coordinate and carbon oxidation state (OS_C_). The solid, dashed-dotted, and dashed lines are a fitted line of ACPs with log(C*), 95% confidence bands, and prediction bands, respectively. (**B**) Mass distributions of VOCs across the three sinks of indoor air (blue bars), surface (orange bars), and ventilation (red bars) vary with *K*_oa_ at the ADP time. The blue dots are mass ratios of selected VOCs in the gas phase. (**C**) Observed log(*K*_sa_) (red squares and circles) assuming that the surface film is 100 and 10 nm, respectively, and the estimated diffusive depth (blue triangles) assuming that *K*_sa_ = *K*_oa_ and *S/V* = 4 m^−1^.

Measured ACP time scales increase inversely with predicted log(C*) values (Fig. 2A) and vary from 3.2 to 21.0 hours for 26 investigated VOCs. We used the estimation programs interface (U.S. Environmental Protection Agency) suite to determine vapor pressures (and therefore C*) of inferred speciated compounds. On the basis of their volatilities, these investigated VOCs fall into the classes of VOCs and intermediate VOCs (IVOCs) (*48*). We calculated the carbon oxidation state (OS_C_) as 2O/C − H/C, where O/C and H/C are the oxygen-to-carbon and hydrogen-to-carbon ratios of a given compound, respectively. Although the isomeric structures of VOCs are not resolved by our instruments, we evaluate the VOC chemical structure based on proton transfer reaction (PTR) ionization preference (*49*) and the most probable species in North America wildfires (*7*, *8*). Detailed information for this figure is in table S1. The variability of ACP values suggests that the gas-surface partitioning process is influenced by vapor pressures. For furan (C_4_H_4_O) (C* = 2.2 × 10^9^ μg m^−3^), the surface starts to serve as a net emission source at ACP = 4.4 hours, whereas for C_8_H_8_O_2_ (C* = 2.1 × 10^4^ μg m^−3^), the ACP is 16.3 hours. For continuous wildfire smoke infiltration, indoor surface reservoirs will more effectively act as a short-term passive removal mechanism for lower volatility species than for higher volatility ones. Assuming that this linear trend of ACP with C* holds for lower vapor pressure compounds, we predict that for a semivolatile VOC (SVOC; with C* = 0.3 μg m^−3^), the ACP will be 26.3 hours, and for an extremely low volatility VOC (with C* = 3 × 10^−5^ μg m^−3^), the ACP will be 35.3 hours. Overall, these data show that indoor surfaces will serve as a net emission source for most VOCs just 1day after the outdoor air pollution diminishes, acknowledging that ACP will decrease at faster ventilation rates. Thus, while the surface is a short-term passive absorbent, it will become a longer-term source of smoke VOCs, on the order of a day after the outdoor pollution source has stopped. Chemical structure influences partitioning time scales. More oxidized and functionalized compounds with higher OS_C_ tend to have longer ACP time scales, implying lower C*; larger octanol air partition coefficients (*K*_oa_); and, at least initially, more-efficient removal from air by indoor surfaces. The impact of oxidation state on ACP is more obvious while plotting ACP with molar mass and OS_C_ (fig. S10A). The relationship between ADP and log(C*) is not apparent (fig. S10B), because most ADP values are constrained to a narrow time scale of 1.5 to 3.5 hours after smoke addition when the concentration decay rate is influenced by the coupled processes of gas diffusion, indoor air mixing, and gas-surface partitioning.

The fate of smoke VOCs indoors includes exfiltration to outdoor air by ventilation, partitioning to surface reservoirs, and remaining in (or repartitioning to) indoor air. Figure 2B summarizes these fates as the mass distribution ratio as a function of *K*_oa_ at the ADP time point (i.e., when surface adsorption and desorption rates are equal), as calculated following section S5. *K*_oa_ values are estimated from C* values (*50*). More volatile compounds are more readily exfiltrated via ventilation and thus affect outdoor urban air more than less volatile compounds. However, at ADP time scales, the effect of ventilation is limited for all VOCs when the test house operated with ACR = 0.24 hours^−1^, and even for the most volatile species with *K*_oa_ < 3 (e.g., C_4_H_4_O and acrolein), the average mass ratio for ventilation is 0.27, smaller than the ratio of 0.36 in surface sink. For less volatile VOCs with *K*_oa_ > 7 (e.g., 2-hydroxymethyl phenol and glutaric acid), the mass ratio in the surface sink increases to ~0.8 at the ADP. This trend implies that most smoke VOCs have enough time to reach adsorption-desorption equilibrium before they are dominantly ventilated out of the house, demonstrating the reduced role of house ventilation on removing smoke VOCs. We probe the temporal evolution of this equilibrium by calculating smoke VOC fate at the ACP time (fig. S11B). At this longer time point, ventilation becomes less important as a sink for less volatile, higher *K*_oa_ compounds. By this time, 50.0% of the infiltrated C_4_H_4_O will ventilate outdoors by the ACP (7.2 hours), whereas only 17.3% of infiltrated C_8_H_8_O_2_ will ventilate by its ACP (16.3 hours). As the mass ratio in the ventilation sink is based on an average VOC concentration in the house, we tested the sensitivity of the model by assuming a maximum ventilation scenario (fig. S11, C and D) in which we use the upper limit of the VOC concentrations for the ventilation flow. The VOC concentration deviation is due to the single-point injection and resulting concentration gradient, and this deviation disappears after 2.5 hours when the house air is mixed well by the indoor recirculation and ventilation system (section S2). In this high ventilation case, the mass distribution ratio of the ventilation sinks increases only slightly (on average, +0.06), and the trends with *K*_oa_ remain consistent.

Partitioning smoke VOCs from the air to a surface contributes to the development of surface reservoirs. The surface-air equilibrium coefficient, *K*_sa_, is a parameter that describes the ratio of VOC concentration in the condensed phase versus gas phase. We calculate *K*_sa_ values of the investigated smoke VOCs by using the equation below (section S5)

$$K_{\mathrm{sa}}=\frac{W(t_{e})\cdot (V/S)}{G(t_{e})\cdot L}$$ (3)

where *W*(*t_e_*)/*G*(*t_e_*) is the derived/measured mass distribution between surface sink and gas sink at ADP time, *V*/*S* is the indoor volume surface ratio, and *L* is the assumed diffusive depth into indoor surfaces. Assuming a diffusive depth of 100 nm (Fig. 2C, red squares), predicted *K*_sa_ values match *K*_oa_ for VOCs with lower volatility (i.e., *K*_oa_ > 7); however, *K*_sa_ values are consistently higher than *K*_oa_ for more volatile compounds. This discrepancy is enhanced when assuming a diffusive depth of 10 nm (Fig. 2C, red circles). These estimations imply that smoke VOCs exhibit gas-surface partitioning behavior that aligns with much lower volatilities than otherwise expected. This semivolatile behavior of VOCs is consistent with previous observations (*19*) and highlights the challenge of modeling indoor surface films (*23*, *28*, *29*). Hypotheses to explain this enhanced surface adsorption include the extended internal surface areas of permeable materials such as painted walls and coated wood (*22*, *26*, *51*), the effect of diffusion of VOCs into various surface reservoirs at compound-specific depths (*26*), and chemical reactions between adsorbed components or between an adsorbed gas and the material surface (*23*).

To explore the potential for smoke VOCs diffusing to different depths into indoor surfaces, we model this depth using the equilibrium surface/gas mass ratio (*W_i_*/*G_i_* at ADP) and assuming a *V*/*S* = 1/4 m^−1^ and *K*_sa_ = *K*_oa_. The derived diffusive depth *L* varies from 10 to 10^6^ nm for VOCs with different *K*_oa_ values as blue triangles in Fig. 2C. We note that *L* is the diffusive depth into an equivalent organic film with partitioning coefficient close to *K*_oa_, while, in reality, molecules are diffusing into the surface films and the underlying bulk materials. Thus, on short time scales of a few hours, smaller molecules with higher diffusion coefficients would diffuse farther into the surface materials, whereas larger molecules become more concentrated near the surface-air boundary layer, resulting in a thinner estimated diffusive depth (*26*, *52*). The multicomponent surface film would thus have a single physical depth from building material surface to air but with compound-specific concentration gradients. The upper value of diffusive depth (10^6^ nm or 1 mm) is much larger than a typical organic film thickness of 100 nm, implying that VOCs can potentially diffuse into deeper surface reservoirs such as paint or other porous materials. The abundance of gas compounds in surface reservoirs confirms the important role of surface reservoirs in controlling air concentrations of many indoor air constituents (*19*) and, more importantly, explains why smoke VOCs persist in indoor air for such long periods of time even if introduced to the building for only a few minutes or hours. The fate and potential persistence of smoke VOCs and other indoor air pollutants depend on chemical structure and properties, namely, the *K*_oa_ and volatility.

### Surface cleaning removes smoke VOC reservoirs

**Fig. 3.**
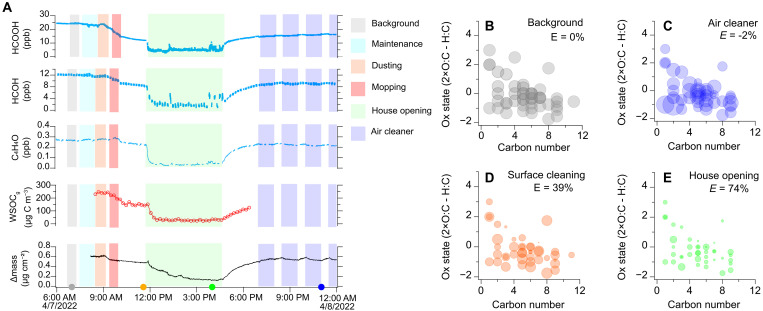
VOC concentration changes in response to different indoor activities. (**A**) Concentration changes for three exemplary VOCs, WSOC_g_, and surface mass changes monitored by a quartz crystal microbalance coated with a porous TiO_2_ thin film. The circles on the *x* axis show the time points of (B) to (E) with colors matching each activity. (**B** to **E**) Mass ratios of 43 investigated VOCs relative to the background case for different activities as a function of carbon number ands. The area of each solid circle represents the ratio of the measured concentration over the background concentration for the (B) background, (C) 1 hour after running air cleaners, (D) 1.5 hours after surface cleaning activities, and (E) 4 hours after opening all windows and doors.

The dominance of indoor surface reservoirs in controlling air concentrations of smoke VOCs raises the question of whether removing these reservoirs can reduce air concentrations and thus human exposure in indoor environments. To investigate removal methods, we conducted multiple house activities, including dusting, mopping, house opening, and running air cleaners, after the smoke additions. These activities consistently affected concentrations of a carboxylic acid [formic acid HCOOH)], a known indoor air toxic [formaldehyde (HCOH)], a volatile smoke tracer (C_4_H_4_O), and WSOC_g_, as well as the mass change (Δmass) of a TiO_2_ thin film–coated quartz surface sample, which provides a proxy measurement for surface films that are driven by partitioning and deposition from indoor air (Fig. 3A). All compounds show consistent responses to perturbations. Background air concentrations and surface reservoirs are stable before cleaning activities, with minor variations at 7:30 a.m., when researchers entered the house to conduct maintenance activities. Between 8:30 and 10:00 a.m., surface cleaning activities included (i) dusting and vacuuming horizontal surfaces and (ii) mopping floors and cleaning kitchen counters with a prepared solution over the 52-m^2^ floor surface and kitchen table surfaces (~40% of the ground area of the first floor). The overall effect of our combined surface cleaning (dusting, vacuuming, and mopping) caused most VOC concentrations to drop by 50.3% (HCOOH), 32.0% (HCOH), and 19.0% (C_4_H_4_O), consistent with substantial removal of the surface reservoir and subsequent loss of the surface emission source. Commercial cleaning solutions do release specific VOC ingredients (*53*), but this analysis focuses on smoke-related VOCs. The removal of the surface reservoir not only suppresses the emission source of those smoke VOCs but also provides clean surfaces to which gases can partition.

The WSOC_g_ represents an integrated measurement of water-soluble gas-phase organic compounds in the house air, while the mass measured on the TiO_2_ thin film–coated quartz surface represents a proxy of total surface reservoir, acknowledging that reservoirs will be material specific. The WSOC_g_ concentration decreased 39.0% in response to surface cleaning, demonstrating the potential power of surface removal of reservoirs for improving indoor air quality. WSOC_g_ decayed at a rate of 0.42 hours^−1^ after surface cleaning, probably representing the combined effects of fast adsorption of gas VOC molecules on the cleaned surface and continued gas desorption from the now smaller uncleaned surfaces. The decay rate decreases as the house reaches a new equilibrium between the now-smaller surface reservoir and unchanged volume of house air. The mass measured by the QCM decreases by 0.128 μg/cm^2^ after house cleaning activities, reflecting the net transfer of VOC molecules from an uncleaned surface into the gas phase. Overall, these observations demonstrate that surface cleaning can reduce the otherwise persistent smoke VOC indoor reservoir and consequent emissions and thus improve indoor air quality following wildfire smoke events.

Outdoor air is typically lower in VOC concentrations than indoor air in the absence of wildfire smoke, nearby pollution sources, or substantial urban smog. Window opening thus presents an opportunity to enhance ventilation, reduce indoor air concentrations, and shift the surface-air equilibrium to favor gas desorption and thereby reduce the magnitude of indoor surface reservoirs. To investigate the effectiveness of window opening, the house was extensively ventilated with all the windows and doors open for several hours. In response to the open house, indoor VOC concentrations in the gas phase dropped markedly (WSOC_g_ decreased 81.6%). The mass on the QCM decreased gradually, indicating that surface reservoir molecules desorbed into the diluted, cleaner air. Assuming a film thickness of 100 nm, the mass loss of 0.336 μg/cm^2^ due to 4.5-hour ventilation only accounts for 3.4% of the sample mass, demonstrating the substantial capacity of surface reservoirs to hold indoor VOCs. However, this initial reduction in indoor air concentrations is only temporary. When the house windows and doors were closed at 4:30 p.m., the air rapidly refilled with repartitioned VOCs to similar concentration levels before house opening. This observation demonstrates the persistence of surface reservoirs for VOCs. In contrast to ventilation, the surface cleaning established a new baseline concentration for smoke VOCs that were far lower than presurface cleaning levels.

Portable air cleaners showed similar time scales (i.e., temporary suppression of VOC levels only during the cleaning activity) but far more limited effects on VOC removal relative to window opening. Figure 3 highlights time points when an air cleaner was operational. This air cleaner used active carbon filters and a plasma system; other commercial air cleaners were tested during the study, but none were more effective than the highlighted one at removing VOCs. While the air cleaner was operational, HCOOH, HCOH, and C_4_H_4_O were removed by 3.8, 3.1, and 4.8%, respectively. Similar to window opening, VOC levels returned to pre-air cleaner concentrations within ~30 min of turning the portable air cleaner off. The effectiveness of the selected air cleaner was limited by low clean air delivery rate (CADR) coupled with the abundant indoor surface reservoirs that continuously emit these VOCs quickly to replenish indoor air. For example, while an air cleaner is operational, the surface emission rate of C_4_H_4_O is 1113 μg/hour (section S13) in competition with a maximum air cleaner removal rate of 54.16 μg/hour. Here, the upper limit of the estimated CADR for VOCs for this applied portable air cleaner was 78 m^3^ hour^−1^. For these smoke VOCs, the CADR would have to be ~20 times greater to compete with the surface reservoir. While there is great variability across portable air cleaners, the house opening experiment described above demonstrates their limitation in removing pollutant VOCs: While air levels may be temporarily suppressed, the surface reservoir is too large for VOC removal from the air to provide long-term decreases in pollutant air concentrations when the window opening or air cleaning activity ends.

To quantify the efficiency of different house activities on removing VOCs from indoor air, we compare changes in the subset of oxidized VOCs detected by the iodide chemical ionization mass spectrometry (CIMS) (Fig. 3B). We quantify the average efficacy of an activity on measured indoor VOCs with an equal-weighted cleaning effectiveness, which we calculate as $E=1-\frac{\sum_{i=1}^{n}S_{i}}{n}$, where *n* is the total number of VOC species, *i* is the index of each VOC, and *S_i_* is the normalized concentration to the background concentration level. The tested air cleaners had no substantial net effect on the investigated VOC suite, either during or after air cleaner operation; a more detailed analysis of air cleaner effectiveness and potential for by-products is the subject of a separate paper. In contrast, dusting and mopping induces a removal effectiveness of 39 ± 5% immediately (2 hours) after cleaning and 38 ± 8% 12 hours after cleaning (fig. S13). Surface cleaning efficiency persists for at least 12 hours and is notably similar to the area ratio of the cleaned surface to the total surface area of the house (40%). Window opening was the fastest and most effective way to reduce indoor VOC levels with *E* = 74 ± 2%. However, window opening benefits are short-lived, with effectiveness dropping to −6 ± 18% after 3 hours (fig. S13). Expanding the VOC suite under study has little effect on these conclusions but demonstrates that the surface cleaning is more effective for more functionalized and more oxidized organic compounds (figs. S14 and S15), whereas house opening activities are equally effective across the broad suite of VOCs. These observations demonstrate that while indoor surface reservoirs affect indoor air levels, the physical removal of these reservoirs offers an air cleaning approach to improve indoor air quality after substantially polluting events.

## DISCUSSION

We show that a wide array of VOCs participates in the dynamic gas-surface partitioning and that indoor surfaces have great partitioning capacity as transient reservoirs for those smoke VOCs. These surface reservoirs lower gas phase VOC concentrations when the house is heavily polluted by outdoor wildfire smoke, thereby reducing acute indoor exposure to those compounds during the outdoor pollution event. In contrast, when pollutant levels in outdoor (and thus indoor) air are subsequently decreased after smoke events, those indoor surface reservoirs become net emission sources to indoor air, presenting a long-term (days to weeks) indoor exposure route for smoke VOCs. Although our measurement only covers classes of VOC and IVOC, this partitioning behavior also holds for smoke SVOC (*K*_oa_ > 10^9^), implying persistence that could last for months to years in the absence of intervention, depending on the properties of the smoke VOCs (*17*). Federal and state guidance for wildfire smoke events focuses on reducing exposure to PM through reductions in infiltration including sealing and positively pressurizing buildings, use of HVAC systems for filtration, and use of portable air cleaners (*14*). California has a new policy requiring improved filtration (MERV 13) for new construction (*54*). However, these activities are not designed to remove smoke VOCs, and little is known about the effectiveness of HVAC on VOC removal. HVAC systems can be sinks for indoor WSOC_g_ via removal to condensate and wet surfaces (*55*, *56*); HVAC filters also have the potential to act as surface reservoirs and thus re-emit VOCs on longer time scales. Our results suggest that increasing ventilation of outdoor air after a smoke event has little effect on the surface reservoir, although it will affect the time scales for surface removal to transition to re-emission. Thus, surface removal by building material surfaces has a short-term suppression of air pollutants but long-term re-emission of chemicals into the gas phase after smoke events.

Despite the complexity of gas-surface partitioning behavior of smoke VOCs, two time scales obtained from real-time data provide a simplified description of the dynamic system: ADP and ACP. We include these two time scales in a model framework that mathematically describes their physical meaning and demonstrates that specific molecular properties govern partitioning in a predictable manner. Smaller ADP values indicate a faster adsorption process. The larger uncertainties associated with ADPs reflect the entangled processes of gas diffusion, mixing, and partitioning. These competing processes mean that a single classic time scale (i.e., residence time or corresponded decay rate) cannot wholly characterize the dynamic gas-surface partitioning processes, even for the first few hours. In contrast, the ACP reflects the gas volatilities (Fig. 2A) and surface/gas mass distributions (fig. S12) without the influence of those initial entangled processes (diffusion, mixing, and partitioning). Further, our analysis of surface-air partitioning coefficients suggests that lighter, more volatile molecules penetrate deeper into indoor surfaces than less volatile compounds (*26*, *52*) and thus maintain higher abundances in the condensed phase than previously expected, explaining why compounds traditionally considered volatile in the outdoor atmosphere exhibit semivolatile behavior indoors (*19*). Enhanced VOC persistence is to be expected if the house is exposed to wildfire smoke for long time periods (weeks to months) that enable VOCs to diffuse deeply into surface reservoirs, or if the house has more fabric or furnishings and thus more effective surface area. Overall, while our study uses wood smoke VOC additions in a single test house to demonstrate the principles and implications of indoor partitioning, our approach should hold for other pollution events, including cooking emissions (*57*), pesticide or commercial chemical product application, and infiltration of outdoor smog or other air pollutants (*58*, *59*).

While the abundant indoor surface reservoirs of organic compounds provide a persistent emission source of smoke VOCs, physical removal by vacuuming and mopping after a smoke event provides a simple, low-cost mechanism to effectively and permanently clean indoor air of previously infiltrated smoke VOCs. The principles of gas-surface partitioning suggest that physical surface cleaning will be effective for different building types, including long-term care facilities and schools, to provide a mechanism for mitigating exposure to smoke VOCs of sensitive groups. Natural ventilation with clean, post-smoke event outdoor air and application of air cleaning devices can temporarily reduce indoor concentrations of smoke VOCs, but the abundance and persistence of surface reservoirs mean that these cleaning methods are limited to only working during active operation of the method (fig. S13). This limitation of ventilation and portable air cleaners for smoke VOCs is distinct from particulate pollution: Ventilation and portable air cleaners can be very effective for removing particles, which do not have continuous emission from surface reservoirs (*38*, *60*). The efficacy of surface cleaning in removing VOC reservoirs will be influenced by both practical and chemical considerations. Practical considerations include the accessibility of surfaces (e.g., hidden or inaccessible areas, fabrics, attics, walls, and ceilings), the choice of cleaning methods (e.g., vacuuming vs mopping), and the physical effort required for manual cleaning. Chemical considerations include the presence of different indoor surfaces (e.g., carpet, fabrics, and painted surfaces) that may influence VOC surface reservoirs and respond disparately to different cleaning approaches. Further, cleaning solutions can be sources of chemical air contaminants of concern (*57*) or even induce additional chemical reactions that create toxic by-products, as is the case for chlorine-based cleaners (*40*, *61*, *62*). These limitations provide opportunities for further research into the efficacy of different types of surface cleaning and development of new surface cleaning technologies.

Wildfires at the wildland-urban interface are increasing across the United States, which our work shows will affect indoor air quality not only during the event but also on longer time scales. We demonstrate the impact of outdoor air pollution on indoor exposure to smoke VOCs and use our experiments to describe the fundamental chemical processes controlling this exposure route. Established methods for reducing indoor levels of PM are not as effective for gaseous air pollutants due to partitioning and the development of a surface reservoir; however, surface cleaning provides a simple, effective solution. Continued efforts to reduce outdoor air pollution and wildfire smoke events are necessary for reducing prolonged exposure to hazardous air pollutants.

## MATERIALS AND METHODS

### Site and experimental design

#### NIST zero-energy house

Measurements were conducted at the National Institute of Standards and Technology’s (NIST) Net-Zero Energy Residential Test Facility (NZERTF), which hosted the Chemical Assessment of Surfaces and Air (CASA) campaign between 2 March and 11 April 2022. CASA was designed to investigate the impact of natural and anthropogenic perturbations (e.g., wildfire smoke, surface/air cleaning, and ozone intrusion) on the indoor air and surface composition through a series of reproducible experiments. The NZERTF is a two-story unfurnished home with four bedrooms and three bathrooms, spanning an area of 242 m^2^ for occupied floors and 243 m^2^ for the unfinished basement and attic, with a total volume of 1484 m^3^ (*41*). The exposed surface area of the entire house is 2170 m^2^; the major surfaces include painted walls (42.1%), wooden floors (11.2%), and painted ceilings (9.7%). The heat recovery ventilation (HRV) system provides an ACR of ~0.2 hours^−1^ by setting an outdoor air flow rate of 250 ± 17 m^3^ hour^−1^. House HVAC recirculation flow rate is set to 1700 m^3^ hour^−1^, corresponding to an indoor recirculation rate of 1.3 hours^−1^. The ventilation system maintained constant conditions throughout the experiment, with the exception of 2 days that were not included in this analysis. The indoor air temperature during the experiments was 24° ± 2°C, with an RH of 30 ± 5%.

#### Smoke addition and cleaning

We used a cocktail smoker (Breville, BSM600SILUSC) to produce smoke from ponderosa pine woodchips. For the direct smoke injections described here, 0.25 to 1.0 g of woodchips were used in each addition. The smoke was rapidly injected into the house within a span of 2 min, and a fan was operated for 10 min to facilitate quick mixing of the smoke with the room air. The bulk chemical composition of the aerosol produced (95% organic, 3% nitrate, 1% sulfate, and 1% ammonium) and VOCs (e.g., formic acid, acetic acids, and formaldehyde) emitted by the cocktail smoker were comparable to those observed in U.S. western wildfires (*7*, *63*). Smoke injections commenced on the afternoon of 21 March, with 23 smoke addition events in the following 2 weeks until 6 April; other experiments that occurred in the house during this time period included ozone additions, whole-house humidification, and the use of various portable air cleaners. Smoke injections included both direct injections of fresh smoke that occurred over 10 min and indirect injections of aged smoke injected from a Teflon chamber following ozone oxidation; indirect injections took around 60 min and were not included in this analysis. During direct smoke injections, submicron organic aerosol concentrations peaked at 100 to 300 μg m^−3^, comparable to levels observed in houses during wildfire smoke events (*10*). Surface cleaning occurred before (21 March) and after (7 April) smoke additions. On 7 April, activities included surface cleaning, house opening (doors and windows), visits by approximately 50 to 60 people during an open house, house closing, and running air cleaners. Surface cleaning activities included approximately 1 hour of dusting and vacuuming on both first and second floors (floor area: 252 m^2^), followed by 0.5 hours of mopping and wiping on the first floor (floor area: 126 m^2^) using a cleaning solution recommended by the Red Cross for smoke odor removal (*64*), which included 7.5 tablespoon of (110.9 ml) trisodium phosphate (TSP), three eight–cups (88.7 ml) of commercial multipurpose cleaner (we selected a fruit-scented, water-based cleaner with key ingredients including sodium dodecylbenzene sulfonate and other sulfates, mixed to manufacturer directions) plus 1.5 gallons (5.7 liters) of water. The commercial air cleaner used on 7 April incorporated a prefilter, high efficiency particulate air filter (HEPA), activated carbon filter, and dual polarity ion technology and caused a more pronounced decrease in indoor VOC levels than other commercial air cleaners that were used but not described here. Detailed descriptions of tested portable air cleaners are in section S13.

### Gas and surface measurements

Gas-phase compounds were measured with two time-of-flight (TOF) CIMSs, one with iodide reagent ions (I-CIMS) and the other with an H_3_O^+^ PTR configuration (PTR-TOF-MS). Details of the ion chemistry (*65*) and operational procedures are described elsewhere (*66*). Experimental details about sampling locations, experimental setup, calibration, background subtraction, inlets, and sensitivity estimates are in section S1. WSOC_g_ was measured semicontinuously in 6-min intervals by sampling room air through a quartz fiber filter (Pall, 47 mm at 25 Lpm) to remove particles, scrubbing particle-free indoor air into liquid water using a mist chamber, and measuring total carbon using an on-line total organic carbon analyzer (Sievers M9 Portable TOC) (*67*–*69*). An Aerodyne TILDAS compact single laser trace gas analyzer detected HCHO (1-s resolution); a cavity ring-down spectroscopy instrument detected CO (Picarro G2401) and a photoacoustic instrument (Innova 1412i) detected SF_6_. Indoor ACR is determined from the decay of SF_6_ and CO concentration following injection. A QCM with a deposited thin TiO_2_ porous film was applied to measure the mass variation of the indoor surface films (*44*, *70*, *71*), and section S1 provides more details of the QCM operation.

## Supplementary Materials

### This PDF file includes:

Sections S1 to S14 Figs. S1 to S17 Tables S1 and S2 References

## References

[1] V. C. Radeloff, D. P. Helmers, H. A. Kramer, M. H. Mockrin, P. M. Alexandre, A. Bar-Massada, V. Butsic, T. J. Hawbaker, S. Martinuzzi, A. D. Syphard, S. I. Stewart, Rapid growth of the US wildland-urban interface raises wildfire risk. Proc. Natl. Acad. Sci. U.S.A. 115, 3314–3319 (2018). 29531054 10.1073/pnas.1718850115 PMC5879688

[2] S. Hantson, N. Andela, M. L. Goulden, J. T. Randerson, Human-ignited fires result in more extreme fire behavior and ecosystem impacts. Nat. Commun. 13, 2717 (2022). 35581218 10.1038/s41467-022-30030-2 PMC9114381

[3] National Academies of Sciences, Engineering, and Medicine, *The Chemistry of Fires at the Wildland-Urban Interface* (The National Academies Press, 2022), pp. 214. 36657007

[4] R. Aguilera, T. Corringham, A. Gershunov, T. Benmarhnia, Wildfire smoke impacts respiratory health more than fine particles from other sources: Observational evidence from Southern California. Nat. Commun. 12, 1493 (2021). 33674571 10.1038/s41467-021-21708-0 PMC7935892

[5] C. E. Reid, M. Brauer, F. H. Johnston, M. Jerrett, J. R. Balmes, C. T. Elliott, Critical review of health impacts of wildfire smoke exposure. Environ. Health Perspect. 124, 1334–1343 (2016). 27082891 10.1289/ehp.1409277 PMC5010409

[6] C. D. McClure, D. A. Jaffe, US particulate matter air quality improves except in wildfire-prone areas. Proc. Natl. Acad. Sci. U.S.A. 115, 7901–7906 (2018). 30012611 10.1073/pnas.1804353115 PMC6077721

[7] W. Permar, Q. Wang, V. Selimovic, C. Wielgasz, R. J. Yokelson, R. S. Hornbrook, A. J. Hills, E. C. Apel, I.-T. Ku, Y. Zhou, B. C. Sive, A. P. Sullivan, J. L. Collett, T. L. Campos, B. B. Palm, Q. Y. Peng, J. A. Thornton, L. A. Garofalo, D. K. Farmer, S. M. Kreidenweis, E. J. T. Levin, P. J. DeMott, F. Flocke, E. V. Fischer, L. Hu, Emissions of trace organic gases from western U.S. wildfires based on WE-CAN aircraft measurements. J. Geophys. Res. Atmos. 126, e2020JD033838 (2021).

[8] S. J. Prichard, S. M. O'Neill, P. Eagle, A. G. Andreu, B. Drye, J. Dubowy, S. Urbanski, T. M. Strand, Wildland fire emission factors in North America: Synthesis of existing data, measurement needs and management applications. Int. J. Wildland Fire 29, 132–147 (2020).

[9] J. B. Gilman, B. M. Lerner, W. C. Kuster, P. D. Goldan, C. Warneke, P. R. Veres, J. M. Roberts, J. A. de Gouw, I. R. Burling, R. J. Yokelson, Biomass burning emissions and potential air quality impacts of volatile organic compounds and other trace gases from fuels common in the US. Atmos. Chem. Phys. 15, 13915–13938 (2015).

[10] Y. T. Liang, D. Sengupta, M. J. Campmier, D. M. Lunderberg, J. S. Apte, A. H. Goldstein, Wildfire smoke impacts on indoor air quality assessed using crowdsourced data in California. Proc. Natl. Acad. Sci. U.S.A. 118, e2106478118 (2021). 34465624 10.1073/pnas.2106478118 PMC8433518

[11] K. O’Dell, B. Ford, J. Burkhardt, S. Magzamen, S. C. Anenberg, J. Bayham, E. V. Fischer, J. R. Pierce, Outside in: The relationship between indoor and outdoor particulate air quality during wildfire smoke events in western US cities. Environ. Res. Health 1, 015003 (2023).

[12] K. P. Messier, L. G. Tidwell, C. C. Ghetu, D. Rohlman, R. P. Scott, L. M. Bramer, H. M. Dixon, K. M. Waters, K. A. Anderson, Indoor versus outdoor air quality during wildfires. Environ. Sci. Technol. Lett. 6, 696–701 (2019). 32095488 10.1021/acs.estlett.9b00599 PMC7039657

[13] G. Davison, K. K. Barkjohn, G. S. W. Hagler, A. L. Holder, S. Coefield, C. Noonan, B. Hassett-Sipple, Creating clean air spaces during wildland fire smoke episodes: Web summit summary. Front. Public Health 9, 508971 (2021). 33681116 10.3389/fpubh.2021.508971 PMC7928341

[14] T. Javins, G. Robarge, E. G. Snyder, G. Nilsson, S. J. Emmerich, Protecting building occupants from smoke during wildfire and prescribed burn events. ASHRAE J. 63, 38–43 (2021).

[15] National Academies of Sciences, Engineering, and Medicine, *Why Indoor Chemistry Matters* (The National Academies Press, 2022), pp. 190. 35617448

[16] J. P. D. Abbatt, G. C. Morrison, V. H. Grassian, M. Shiraiwa, C. J. Weschler, P. J. Ziemann, How should we define an indoor surface? Indoor Air 32, e12955 (2022). 35104002 10.1111/ina.12955

[17] C. J. Weschler, W. W. Nazaroff, Semivolatile organic compounds in indoor environments. Atmos. Environ. 42, 9018–9040 (2008).

[18] J. P. D. Abbatt, C. Wang, The atmospheric chemistry of indoor environments. Environ. Sci.: Processes Impacts 22, 25–48 (2020). 10.1039/c9em00386j 31712796

[19] C. Wang, D. B. Collins, C. Arata, A. H. Goldstein, J. M. Mattila, D. K. Farmer, L. Ampollini, P. F. DeCarlo, A. Novoselac, M. E. Vance, W. W. Nazaroff, J. P. D. Abbatt, Surface reservoirs dominate dynamic gas-surface partitioning of many indoor air constituents. Sci. Adv. 6, eaay8973 (2020). 32128415 10.1126/sciadv.aay8973 PMC7030931

[20] D. M. Lunderberg, K. Kristensen, Y. Tian, C. Arata, P. K. Misztal, Y. Liu, N. Kreisberg, E. F. Katz, P. F. DeCarlo, S. Patel, M. E. Vance, W. W. Nazaroff, A. H. Goldstein, Surface emissions modulate indoor SVOC concentrations through volatility-dependent partitioning. Environ. Sci. Technol. 54, 6751–6760 (2020). 32379430 10.1021/acs.est.0c00966

[21] M. Sleiman, J. M. Logue, W. T. Luo, J. F. Pankow, L. A. Gundel, H. Destaillats, Inhalable constituents of thirdhand tobacco smoke: Chemical characterization and health impact considerations. Environ. Sci. Technol. 48, 13093–13101 (2014). 25317906 10.1021/es5036333

[22] A. Manuja, J. Ritchie, K. Buch, Y. X. Wu, C. M. A. Eichler, J. C. Little, L. C. Marr, Total surface area in indoor environments. Environ. Sci. Process. Impacts 21, 1384–1392 (2019). 31246204 10.1039/c9em00157c

[23] P. S. J. Lakey, C. M. A. Eichler, C. Y. Wang, J. C. Little, M. Shiraiwa, Kinetic multi-layer model of film formation, growth, and chemistry (KM-FILM): Boundary layer processes, multi-layer adsorption, bulk diffusion, and heterogeneous reactions. Indoor Air 31, 2070–2083 (2021). 33991124 10.1111/ina.12854

[24] Y. Liu, A. G. Bé, V. W. Or, M. R. Alves, V. H. Grassian, F. M. Geiger, Challenges and opportunities in molecular-level indoor surface chemistry and physics. Cell Rep. Phys. Sci. 1, 100256 (2020).

[25] C. Y. Lim, J. P. Abbatt, Chemical composition, spatial homogeneity, and growth of indoor surface films. Environ. Sci. Technol. 54, 14372–14379 (2020). 33156609 10.1021/acs.est.0c04163

[26] L. B. Algrim, D. Pagonis, J. A. de Gouw, J. L. Jimenez, P. J. Ziemann, Measurements and modeling of absorptive partitioning of volatile organic compounds to painted surfaces. Indoor Air 30, 745–756 (2020). 32077147 10.1111/ina.12654

[27] B. L. Deming, P. J. Ziemann, Quantification of alkenes on indoor surfaces and implications for chemical sources and sinks. Indoor Air 30, 914–924 (2020). 32115779 10.1111/ina.12662

[28] A. P. Ault, V. H. Grassian, N. Carslaw, D. B. Collins, H. Destaillats, D. J. Donaldson, D. K. Farmer, J. L. Jimenez, V. F. McNeill, G. C. Morrison, R. E. O'Brien, M. Shiraiwa, M. E. Vance, J. R. Wells, W. Xiong, Indoor surface chemistry: Developing a molecular picture of reactions on indoor interfaces. Chem 6, 3203–3218 (2020). 32984643 10.1016/j.chempr.2020.08.023 PMC7501779

[29] C. J. Weschler, W. W. Nazaroff, Growth of organic films on indoor surfaces. Indoor Air 27, 1101–1112 (2017). 28556424 10.1111/ina.12396

[30] R. B. Jorgensen, O. Bjorseth, B. Malvik, Chamber testing of adsorption of volatile organic compounds (VOCs) on material surfaces. Indoor Air 9, 2–9 (1999). 10195270 10.1111/j.1600-0668.1999.t01-3-00002.x

[31] D. Won, R. L. Corsi, M. Rynes, Sorptive interactions between VOCs and indoor materials. Indoor Air 11, 246–256 (2001). 11761600 10.1034/j.1600-0668.2001.110406.x

[32] B. A. Tichenor, Z. Guo, J. E. Dunn, L. E. Sparks, M. A. Mason, The Interaction of vapour phase organic compounds with indoor sinks. Indoor Air 1, 23–35 (1991).

[33] F. Thevenet, M. Verriele, P. Harb, S. Thlaijeh, R. Brun, M. Nicolas, S. Angulo-Milhem, The indoor fate of terpenes: Quantification of the limonene uptake by materials. Build. Environ. 188, 107433 (2021).

[34] M. Rizk, M. Verriele, S. Dusanter, C. Schoemaecker, S. Le Calve, N. Locoge, Fast sorption measurements of volatile organic compounds on building materials: Part 1-Methodology developed for field applications. Build. Environ. 99, 200–209 (2016). 10.1016/j.dib.2016.01.011 PMC4753388 26937475

[35] B. C. Singer, A. T. Hodgson, T. Hotchi, K. Y. Ming, R. G. Sextro, E. E. Wood, N. J. Brown, Sorption of organic gases in residential rooms. Atmos. Environ. 41, 3251–3265 (2007).

[36] B. C. Singer, K. L. Revzan, T. Hotchi, A. T. Hodgson, N. J. Brown, Sorption of organic gases in a furnished room. Atmos. Environ. 38, 2483–2494 (2004).

[37] D. B. Collins, R. F. Hems, S. M. Zhou, C. Wang, E. Grignon, M. Alavy, J. A. Siegel, J. P. D. Abbatt, Evidence for gas-surface equilibrium control of indoor nitrous acid. Environ. Sci. Technol. 52, 12419–12427 (2018). 30346749 10.1021/acs.est.8b04512

[38] Q. Ye, J. E. Krechmer, J. D. Shutter, V. P. Barber, Y. Li, E. Helstrom, L. J. Franco, J. L. Cox, A. I. H. Hrdina, M. B. Goss, N. Tahsini, M. Canagaratna, F. N. Keutsch, J. H. Kroll, Real-time laboratory measurements of VOC emissions, removal rates, and byproduct formation from consumer-grade oxidation-based air cleaners. Environ. Sci. Technol. Lett. 8, 1020–1025 (2021).

[39] D. B. Collins, D. K. Farmer, Unintended consequences of air cleaning chemistry. Environ. Sci. Technol. 55, 12172–12179 (2021). 34464124 10.1021/acs.est.1c02582

[40] J. M. Mattila, P. S. J. Lakey, M. Shiraiwa, C. Wang, J. P. D. Abbatt, C. Arata, A. H. Goldstein, L. Ampollini, E. F. Katz, P. F. DeCarlo, S. Zhou, T. F. Kahan, F. J. Cardoso-Saldana, L. H. Ruiz, A. Abeleira, E. K. Boedicker, M. E. Vance, D. K. Farmer, Multiphase chemistry controls inorganic chlorinated and nitrogenated compounds in indoor air during bleach cleaning. Environ. Sci. Technol. 54, 1730–1739 (2020). 31940195 10.1021/acs.est.9b05767

[41] D. Poppendieck, M. Y. Gong, S. Zimmerman, L. Ng, Evaluation of a four-zone indoor exposure model for predicting TCPP concentrations in a low-energy test house. Build. Environ. 199, 107888 (2021). 10.1016/j.buildenv.2021.107888 PMC10947393 38500674

[42] A. H. Fanney, W. Healy, V. Payne, J. Kneifel, L. Ng, B. Doughety, T. Ullah, F. Omar, Small changes yield large results at NIST's net-zero energy residential test facility. J. Sol. Energy Eng. Trans. ASME 139, 061009 (2017). 10.1115/1.4037815 PMC5865221 29581649

[43] R. Habre, D. C. Dorman, J. Abbatt, W. P. Bahnfleth, E. Carter, D. Farmer, G. Gawne-Mittelstaedt, A. H. Goldstein, V. H. Grassian, G. Morrison, J. Peccia, D. Poppendieck, K. A. Prather, M. Shiraiwa, H. M. Stapleton, M. Williams, M. E. Harries, Why indoor chemistry matters: A national academies consensus report. Environ. Sci. Technol. 56, 10560–10563 (2022). 35833728 10.1021/acs.est.2c04163 PMC9352310

[44] H. Schwartz-Narbonne, D. J. Donaldson, Water uptake by indoor surface films. Sci. Rep. 9, 11089 (2019). 31366971 10.1038/s41598-019-47590-x PMC6668427

[45] G. Rubasinghege, V. H. Grassian, Role(s) of adsorbed water in the surface chemistry of environmental interfaces. Chem. Commun. 49, 3071–3094 (2013). 10.1039/c3cc38872g 23417201

[46] W. J. Wei, C. Mandin, O. Ramalho, Influence of indoor environmental factors on mass transfer parameters and concentrations of semi-volatile organic compounds. Chemosphere 195, 223–235 (2018). 29268180 10.1016/j.chemosphere.2017.12.072

[47] K. Kristensen, D. M. Lunderberg, Y. J. Liu, P. K. Misztal, Y. L. Tian, C. L. Arata, W. W. Nazaroff, A. H. Goldstein, Sources and dynamics of semivolatile organic compounds in a single-family residence in northern California. Indoor Air 29, 645–655 (2019). 31004533 10.1111/ina.12561

[48] N. M. Donahue, J. H. Kroll, S. N. Pandis, A. L. Robinson, A two-dimensional volatility basis set - Part 2: Diagnostics of organic-aerosol evolution. Atmos. Chem. Phys. 12, 615–634 (2012).

[49] B. Yuan, A. R. Koss, C. Warneke, M. Coggon, K. Sekimoto, J. A. de Gouw, Proton-transfer-reaction mass spectrometry: Applications in atmospheric sciences. Chem. Rev. 117, 13187–13229 (2017). 28976748 10.1021/acs.chemrev.7b00325

[50] H. Xiao, F. Wania, Is vapor pressure or the octanol-air partition coefficient a better descriptor of the partitioning between gas phase and organic matter? Atmos. Environ. 37, 2867–2878 (2003).

[51] R. Meininghaus, E. Uhde, Diffusion studies of VOC mixtures in a building material. Indoor Air 12, 215–222 (2002). 12532753 10.1034/j.1600-0668.2002.01131.x

[52] M. Shiraiwa, U. Poschl, Mass accommodation and gas-particle partitioning in secondary organic aerosols: Dependence on diffusivity, volatility, particle-phase reactions, and penetration depth. Atmos. Chem. Phys. 21, 1565–1580 (2021).

[53] C. Arata, P. K. Misztal, Y. L. Tian, D. M. Lunderberg, K. Kristensen, A. Novoselac, M. E. Vance, D. K. Farmer, W. W. Nazaroff, A. H. Goldstein, Volatile organic compound emissions during HOMEChem. Indoor Air 31, 2099–2117 (2021). 34272904 10.1111/ina.12906

[54] California Energy Commission, *Buiding Energy Efficiency Standards for Residential and Nonresidential Buildings* (California Energy Commission, 2018).

[55] S. M. Duncan, S. Tomaz, G. Morrison, M. Webb, J. Atkin, J. D. Surratt, B. J. Turpin, Dynamics of residential water-soluble organic gases: Insights into sources and sinks. Environ. Sci. Technol. 53, 1812–1821 (2019). 30633495 10.1021/acs.est.8b05852 PMC7279883

[56] H. Schwartz-Narbonne, J. P. D. Abbatt, P. F. DeCarlo, D. K. Farmer, J. M. Mattila, C. Wang, D. J. Donaldson, J. A. Siegel, Modeling the removal of water-soluble trace gases from indoor air via air conditioner condensate. Environ. Sci. Technol. 55, 10987–10993 (2021). 34342979 10.1021/acs.est.1c02053

[57] A. L. Hodshire, E. Carter, J. M. Mattila, V. Ilacqua, J. Zambrana, J. P. D. Abbatt, A. Abeleira, C. Arata, P. F. DeCarlo, A. H. Goldstein, L. H. Ruiz, M. E. Vance, C. Wang, D. K. Farmer, Detailed investigation of the contribution of gas-phase air contaminants to exposure risk during indoor activities. Environ. Sci. Technol. 56, 12148–12157 (2022). 35952310 10.1021/acs.est.2c01381 PMC9454252

[58] J. M. Mattila, C. Arata, A. Abeleira, Y. Zhou, C. Wang, E. F. Katz, A. H. Goldstein, J. P. D. Abbatt, P. F. DeCarlo, M. E. Vance, D. K. Farmer, Contrasting chemical complexity and the reactive organic carbon budget of indoor and outdoor air. Environ. Sci. Technol. 56, 109–118 (2021). 34910454 10.1021/acs.est.1c03915

[59] D. J. Price, D. A. Day, D. Pagonis, H. Stark, L. B. Algrim, A. V. Handschy, S. Liu, J. E. Krechmer, S. L. Miller, J. F. Hunter, J. A. de Gouw, P. J. Ziemann, J. L. Jimenez, Budgets of organic carbon composition and oxidation in indoor air. Environ. Sci. Technol. 53, 13053–13063 (2019). 31652057 10.1021/acs.est.9b04689

[60] B. Stephens, E. T. Gall, M. Heidarinejad, D. K. Farmer, Interpreting air cleaner performance data. ASHRAE J. 64, 20–30 (2022).

[61] C. Wang, B. Bottorff, E. Reidy, C. M. F. Rosales, D. B. Collins, A. Novoselac, D. K. Farmer, M. E. Vance, P. S. Stevens, J. P. D. Abbatt, Cooking, bleach cleaning, and air conditioning strongly impact levels of HONO in a house. Environ. Sci. Technol. 54, 13488–13497 (2020). 33064464 10.1021/acs.est.0c05356

[62] A. Moravek, T. C. VandenBoer, Z. Finewax, D. Pagonis, B. A. Nault, W. L. Brown, D. A. Day, A. V. Handschy, H. Stark, P. Ziemann, J. L. Jimenez, J. A. de Gouw, C. J. Young, Reactive chlorine emissions from cleaning and reactive nitrogen chemistry in an indoor athletic facility. Environ. Sci. Technol. 56, 15408–15416 (2022). 36326040 10.1021/acs.est.2c04622

[63] L. A. Garofalo, M. A. Pothier, E. J. T. Levin, T. Campos, S. M. Kreidenweis, D. K. Farmer, Emission and evolution of submicron organic aerosol in smoke from wildfires in the Western United States. ACS Earth Space Chem. 3, 1237–1247 (2019).

[64] American National Red Cross, *Cleaning Up After a Fire* (American National Red Cross, 2022); www.redcross.org/get-help/how-to-prepare-for-emergencies/types-of-emergencies/fire/cleaning-up-after-fire.html.

[65] B. H. Lee, F. D. Lopez-Hilfiker, C. Mohr, T. Kurtén, D. R. Worsnop, J. A. Thornton, An iodide-adduct high-resolution time-of-flight chemical-ionization mass spectrometer: Application to atmospheric inorganic and organic compounds. Environ. Sci. Technol. 48, 6309–6317 (2014). 24800638 10.1021/es500362a

[66] P. Brophy, D. K. Farmer, A switchable reagent ion high resolution time-of-flight chemical ionization mass spectrometer for real-time measurement of gas phase oxidized species: characterization from the 2013 southern oxidant and aerosol study. Atmos. Meas. Tech. 8, 2945–2959 (2015).

[67] S. Tomaz, T. Q. Cui, Y. Z. Chen, K. G. Sexton, J. M. Roberts, C. Warneke, R. J. Yokelson, J. D. Surratt, B. J. Turpin, Photochemical cloud processing of primary wildfire emissions as a potential source of secondary organic aerosol. Environ. Sci. Technol. 52, 11027–11037 (2018). 30153017 10.1021/acs.est.8b03293

[68] S. M. Duncan, K. G. Sexton, B. J. Turpin, Oxygenated VOCs, aqueous chemistry, and potential impacts on residential indoor air composition. Indoor Air 28, 198–212 (2018). 28833580 10.1111/ina.12422 PMC5745158

[69] C. J. Hennigan, M. H. Bergin, A. G. Russell, A. Nenes, R. J. Weber, Gas/particle partitioning of water-soluble organic aerosol in Atlanta. Atmos. Chem. Phys. 9, 3613–3628 (2009).

[70] J. Schuttlefield, H. Al-Hosney, A. Zachariah, V. H. Grassian, Attenuated total reflection Fourier transform infrared spectroscopy to investigate water uptake and phase transitions in atmospherically relevant particles. Appl. Spectrosc. 61, 283–292 (2007). 17389068 10.1366/000370207780220868

[71] J. D. Schuttlefield, D. Cox, V. H. Grassian, An investigation of water uptake on clays minerals using ATR-FTIR spectroscopy coupled with quartz crystal microbalance measurements. J. Geophys. Res-Atmos. 112, D21303 (2007).

[72] A. H. Fanney, V. Payne, T. Ullah, L. Ng, M. Boyd, F. Omar, M. Davis, H. Skye, B. Dougherty, B. Polidoro, W. Healy, J. Kneifel, B. Pettit, Net-zero and beyond! Design and performance of NIST's net-zero energy residential test facility. Energ. Buildings 101, 95–109 (2015).

[73] L. C. Ng, S. Zimmerman, J. Good, B. Toll, S. J. Emmerich, A. K. Persily, Estimating real-time infiltration for use in residential ventilation control. Indoor Built Environ. 29, 508–526 (2020). 10.1177/1420326x19870229 PMC7047832 32116470

[74] P. Brophy, D. K. Farmer, Clustering, methodology, and mechanistic insights into acetate chemical ionization using high-resolution time-of-flight mass spectrometry. Atmos. Meas. Tech. 9, 3969–3986 (2016).

[75] F. D. Lopez-Hilfiker, S. Iyer, C. Mohr, B. H. Lee, E. L. D'Ambro, T. Kurten, J. A. Thornton, Constraining the sensitivity of iodide adduct chemical ionization mass spectrometry to multifunctional organic molecules using the collision limit and thermodynamic stability of iodide ion adducts. Atmos. Meas. Tech. 9, 1505–1512 (2016).

[76] J. E. Krechmer, D. Pagonis, P. J. Ziemann, J. L. Jimenez, Quantification of gas-wall partitioning in teflon environmental chambers using rapid bursts of low-volatility oxidized species generated in situ. Environ. Sci. Technol. 50, 5757–5765 (2016). 27138683 10.1021/acs.est.6b00606

[77] U. Pöschl, M. Shiraiwa, Multiphase chemistry at the atmosphere-biosphere interface influencing climate and public health in the anthropocene. Chem. Rev. 115, 4440–4475 (2015). 25856774 10.1021/cr500487s

[78] U. Pöschl, Y. Rudich, M. Ammann, Kinetic model framework for aerosol and cloud surface chemistry and gas-particle interactions–Part 1: General equations, parameters, and terminology. Atmos. Chem. Phys. 7, 5989–6023 (2007).

