# Supplementary material for: The persistence of smoke VOCs indoors: Partitioning, surface cleaning, and air cleaning in a smoke-contaminated house
Source: Sci Adv. 2023 Oct 13;9(41):eadh8263. doi: 10.1126/sciadv.adh8263 (PMC10575580; doi:10.1126/sciadv.adh8263)
Supplement: Supplementary file 1 — Sections S1 to S14 Figs. S1 to S17 Tables S1 and S2 References [file sciadv.adh8263_sm.pdf]

Science Advances
AAAS

Supplementary Materials for

The persistence of smoke VOCs indoors: Partitioning, surface cleaning, and air cleaning in a smoke-contaminated house

Jienan Li *et al.*

Corresponding author: Delphine K. Farmer, delphine.farmer@colostate.edu;
Jienan Li, jienan.li@colostate.edu

Sci. Adv. **9**, eadh8263 (2023)
DOI: 10.1126/sciadv.adh8263

This PDF file includes:

Sections S1 to S14
Figs. S1 to S17
Tables S1 and S2
References

S1 Experimental setup in the NIST house

1) Floor plans and facilities location

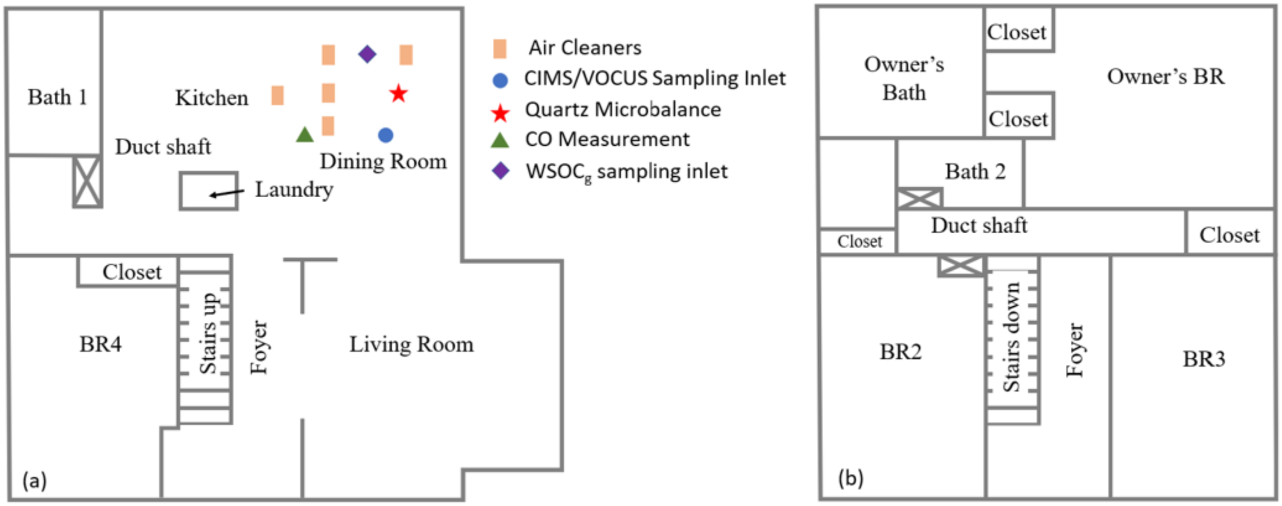

Fig. S1. Floor plans of the NIST zero-energy house, and locations of measuring facilities, inlets, and air cleaners. (a) The first floor. (b) The second floor. A detailed architectural plan can be found at: NZERTF Project Details | NIST (*42, 72, 73*).

2) I-CIMS operation

We measured gas-phase oxygenated VOCs using an Aerodyne high resolution time-of-flight chemical ionization mass spectrometer equipped with the iodide (I^-) reagent ion (I-CIMS). The I-CIMS and PTR-MS sampled outdoors for 5 minutes each hour while sampling indoors the rest of the time. The instrument inlet was positioned in the dining room at a height of ~2 m. A 30.5 m heated 6.35 mm PFA (perfluoroalkoxy alkane) line was used with a flow rate of 5.0 Lpm. We increased the sampling flow rate to 11.5 Lpm in certain experiments to minimize residence time, especially when prioritizing observations of less-volatile species. Ambient air draws through a critical orifice at 1.9 standard liters per minute (slpm) into the ion molecule reactor (IMR), which is maintained at a pressure of 100 mbar (1mbar = 100 Pa). Iodide ions are generated by passing a 1 slpm flow of ultra-high purity (UHP) N_2 over a permeation tube filled with methyl iodide and then through the Po-210 ion source into the IMR. To minimize the effect of humidity on instrument sensitivity, the relative humidity (RH) in the indoor environment was controlled at 30 % ± 5 %. The impact of humidity on the measurement is further reduced by introducing a 0.8 slpm flow of high humidity N_2 through a third port into the IMR and optimally balancing the ratio of $I(H_2O)^-/I^-$ ions to approximately 1/3. The instrument background signal is established by routinely introducing UHP N_2 directly in front of the critical orifice to displace the incoming ambient air. To ensure that the RH of this N_2 flow the same as the sample air, the humidity system functions at a constant flow and adjusts the RH using two mass flow controllers (MKS Instruments, Inc. 1179A) and a PID loop that controls the RH based on readings from an inline RH sensor (Omega Engineering HX71-V1). The total flow rate from the two mass flow controllers is held constant by the PID control loop, and the ratio of the two flows can be varied to generate a wide range of humidified air (0 to 90 %) (*74*).

Throughout the campaign, we evaluated the performance of the instrument, including its baseline, single ion area, threshold, and mass resolution, to ensure consistency. The sensitivity of the I-CIMS was determined through calibration with authentic standards of C1-C5 carboxylic acids, which varied in sensitivity by less than 20 % throughout. We detected compounds using exact mass and relative ion abundance and only considering ionization with I^- (excluding water clusters). However, for most CIMS-identified compounds, except for C1-C5 carboxylic acids, authentic standards were not utilized for positive identification and quantification due to the complexity of biomass burning emissions and the potential presence of multiple isomers with varying ionization efficiencies. We determined the concentration of $C_6H_6O_3$ clustered with iodide using its dV50 and the sensitivity curve obtained after the CASA campaign, following procedures described by Mattila et al (*40, 75*). We use Tofware (v 2.5.7, Tofwerk) and Igor (v 7.08, Wavemetrics) to analyze data. We normalize each time trace to the total reagent ion signal of $[I(H_2O)^- + I^-]$ and subtract a background signal.

Several smoke VOCs required a time-varying background for a few hours following the smoke injection. We corrected for this background by generating a fit to the background samples. As an example, Fig. S2a shows the varying signal of $C_6H_6IO_3^-$ following injection. The periodic background samples (observed as dips in in the red decay curve when the IMR was flooded with UHP N_2) show a clearly varying background signal, which we hypothesize is due to adsorption of $C_6H_6O_3$ to the walls of the IMR. Our inlet zeroing experiment supports this hypothesis by revealing that as the compounds become sticker, the sorption to inlet tubing becomes less important because the gas-wall adsorption portioning of the IMR starts to become rate-limiting process. This finding is consistent with the observation in a previous study (*76*). To obtain a smooth decay shape, we determine a fitted background line to correct for this IMR sink effect using reference points that were set at 45 seconds after IMR was filled with pure nitrogen. Fig. S2b shows that the corrected gas phase concentration of $C_6H_6O_3$ was lower than the CIMS observed value and the decay trend is steeper. This fitted line was only applied to the $C_6H_6O_3$ time trace during the first 6 hours following smoke injection when the curve decays quickly. After 6 hours, when the signal decay is relatively flat, the background subtraction followed the more standard routine of subtracting the value of the reference point in each hour from the timeseries.

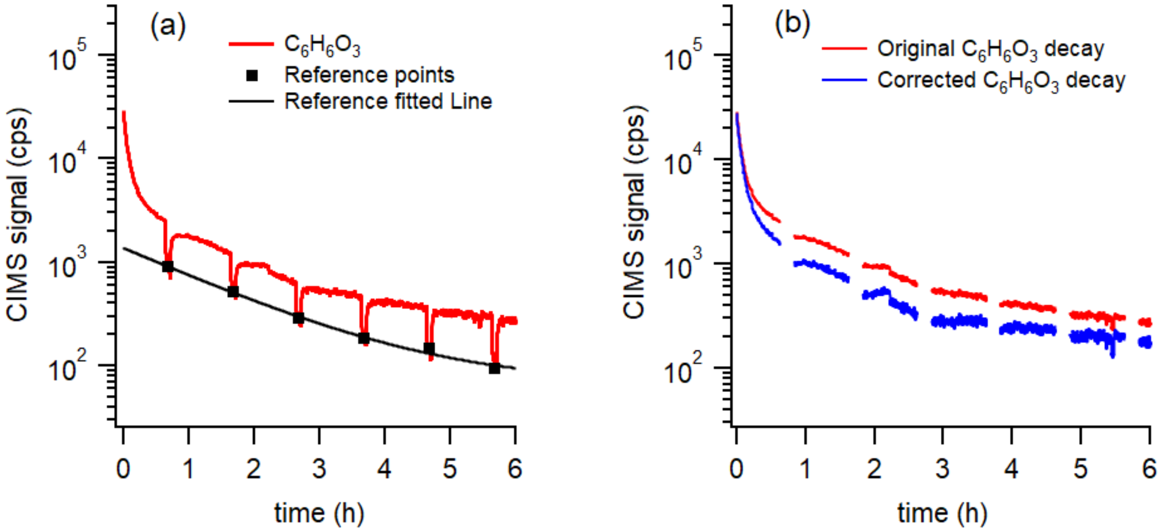

Fig. S2. (a) The red line is the original $C_6H_6IO_3^-$ signal decay with time. The black line shows the fitted background line with reference points (black squares) for the background subtraction. (b)

By subtracting this fitted background line from the original signal (red), we derive a corrected signal (blue) that accounts for this varying background.

3) PTR-MS operation

A VOCUS 2R PTR-ToF-MS was operated during CASA to measure a population of VOC carbon the I^- CIMS is not sensitive to such as monofunctional ketones and aldehydes, heterocycles (including furan), and monoterpenes. The instrument sub-sampled, at 100 mLpm, from a 30.5 m heated 6.1 mm PFA (perfluoroalkoxy alkane) line with a flowrate of ~5 Lpm. Hourly zeros and calibrations were performed using ultra zero air and a standard cylinder of 14 different VOCs (Apel-Riemer). We measured a campaign median sensitivity for furan, $C_4H_4OH^+$ (m/z 69.034), of 760 cps ppb_v^{-1} ± 120 cps ppb_v^{-1}. Average mass resolution was ~10500 (m/Δm). The BSQ voltage was set to 380 V and consequently the sensitivity to m/z below ~m/z 57 was low (i.e., ethanol, acetonitrile, formic acid, ect.). The PTR-MS sampled with an extraction frequency of 14.9 kHz (m/z range 4 to 599). All data processing including mass calibration and high-resolution peak fitting were performed in Tofware v3.2.5.

4) Quartz crystal microbalance (QCM) operation

A quartz crystal microbalance (QCM) technique was used for direct and quantitative estimation of the surface adsorption process (*70, 71*). A commercial QCM (QCM200, Stanford Research Systems) consists of a piezoelectric oscillator and 25.4 mm-diameter gold/Cr polished quartz crystal with fundamental frequency of 5 MHz. Mass loading on a geometrically small quartz crystal surface with little porosity is small and in the order of long-term drifts. To overcome this limitation, in this study, a thin film of titanium dioxide (rutile-TiO_2, BET surface area of 17 m^2 g^{-1} ± 1 m^2 g^{-1}) particles was coated onto the quartz crystal to better mimic a porous film with higher surface area of the indoor surface films. This increase in surface area was also useful for offsetting small drifts in the baseline due to larger mass loading onto the higher surface area and greater adsorption capacity of the TiO_2 porous film. These thin films were prepared by drop-casting ~500 μg/mL of TiO_2 suspension in methanol onto the active area of the crystal (0.4 cm^2). The frequency of the crystal with dry film was then measured to determine the exact mass of the thin film. Typically, thin film masses were in the range of 300 μg to 400 μg. A maximum mass load of < 500 μg is used in order to maintain a linear frequency-mass response in crystals where the maximum frequency shift for these crystals needs to be 1 % of the fundamental frequency. The thin film coated crystal was finally placed in the test house in the horizontal position with crystal surface facing upwards to directly measure mass variation. These surfaces are representative of the side walls and ceiling, but not the floor. All the mass changes (Δmass μg cm^{-2}) of the TiO_2 thin film coated quartz surface sample were calculated based on the active geometrical area of the crystal.

S2 The calculation of air change rate (ACR) and the correction of the gas phase dilution due to indoor air mixing

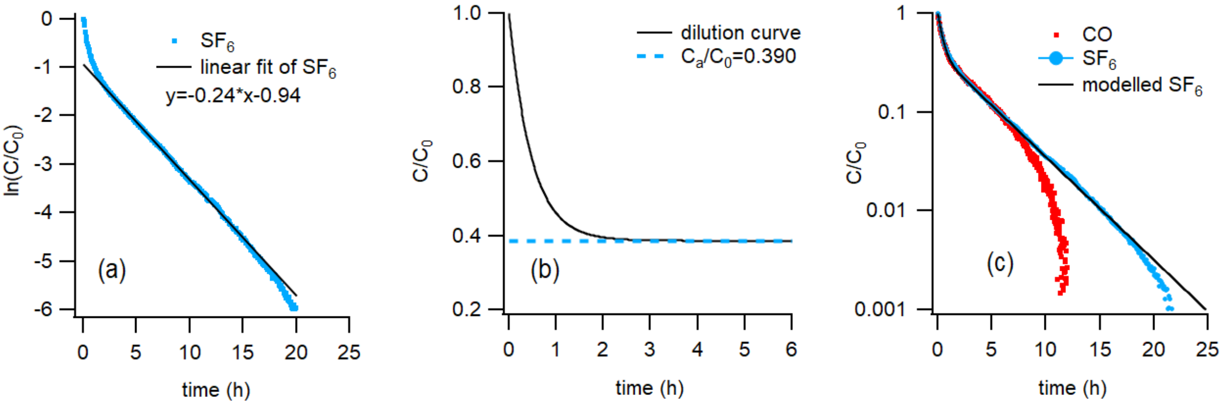

Fig. S3. (a) We estimate ACR from the concentration decay of SF_6 following periodic injection. The linear fit of the SF_6 decay is taken between 3 and 16 hours. The ACR is determined as the absolute value of the fitted slope with a unit of h^{-1}. (b) Indoor recirculation creates a dilution effect on initial gas concentrations decays, which we model assuming no partitioning, zero ACR, and a house recirculation rate of 1.3 h^{-1}. (c) Modelled SF_6 decay is consistent with the observed SF_6 and CO decay. Deviations between modeled and measured values occur when observed concentrations reach the indoor CO background level and the SF_6 detection limit, respectively.

We used sulfur hexafluoride (SF_6) as a tracer gas to estimate air change rate (ACR). SF_6 was simultaneously injected into the house via six distributed locations. A linear relationship was observed between 3 hours and 18 hours as indicated by the black line in Fig. S3a, and the derived ACR for ventilation is 0.24 h^{-1}. The linearity observed indicates that the house ventilation system was well-controlled during smoke addition events. The non-linear SF_6 decay during the initial 2.5 hours is driven by a combination of this ACR (0.24 h^{-1}) and the indoor HVAC recirculation rate (RR) of 1.3 h^{-1} as seen in Fig. S3b. Assuming no ventilation and negligible SF_6 surface partitioning, the CASA house would take around 2.5 hours for SF_6 to be well mixed (< 1 % difference) given a RR = 1.3 h^{-1}. Therefore, the observed initial SF_6 concentration is overestimated by ~2.6 (= C_0/C_a) times when compared to the average well-mixed concentration. By applying a combined effect of the ACR and RR, the modelled SF_6 concentration matches well with the monitored values as seen in Fig. S3c. This dilution effect results from the indoor recirculation that needs to be corrected when evaluating the VOC partitioning behavior by assuming the house air is well-mixed, as discussed below.

Carbon monoxide (CO) is a smoke tracer for estimating the dilution of smoke concentration due to its simultaneous emissions with VOCs (at least in minutes time scale) and its minimal surface adsorption. By comparing the decay of CO to that of SF_6 at the initial 6.5 hours in Fig. S3c, we can assume that CO and smoke VOCs experience similar dilution processes after wood burning. The decay of smoke VOCs can then be corrected using the modeled parameters and dilution curve. Additionally, this correction allows for direct comparison between smoke VOCs and modeled SF_6 decay, even though they were not injected simultaneously. However, we note that when CO concentration drops to an indoor background level of ~ 200 ppb, any subsequent ventilation-driven decay will be obscured by variations in ambient CO levels, as shown in the discrepancy between CO and SF_6 decays after 6.5 hours. The similarity between CO and SF_6 suggests that CO may be a promising tracer for future studies as it is a far less potent greenhouse

gas than SF_6, although safety issues of injecting high concentrations of CO must be considered in occupied homes.

We use a simplified two-box model, as seen in Fig. S4a, to correct the dilution effect of indoor recirculation. The gas-phase measurement facilities are placed in region 1, while the remainder of the house is placed in region 2. The volumes of these regions are represented by V and V' respectively. In smoke addition experiments, smoke is injected and evenly distributed within region 1 at time t = 0, before being transported throughout the rest of the house due to indoor recirculation. As an example, we assume that SF_6 is well-mixed in each region at any time with concentrations of C and C', respectively. Q represents the flow rate of between the volumes.

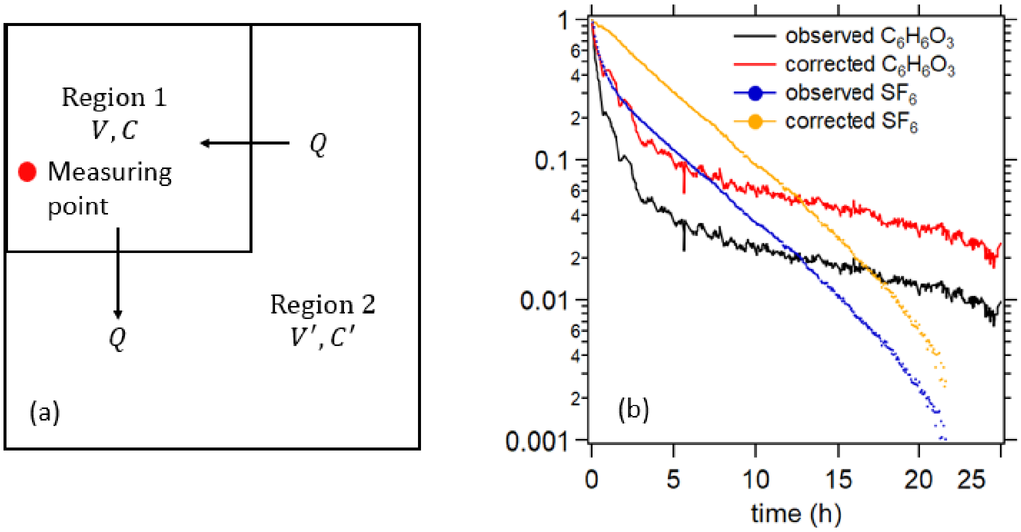

Fig. S4. (a) We use a two-box model to simulate the dilution effect in the test house. The red dot represents the location of the measuring facilities. (b) The normalized concentration decay before and after the correction of the dilution effect due to indoor air mixing.

At t = 0, SF_6 is injected and assumed to be quickly well-mixed in region 1, with an initial concentration $C = C_0$. At this time, C' is equal to 0 assuming a background concentration of SF_6 is not present in the house or has been subtracted. Assuming no surface partitioning or indoor-outdoor ventilation, the SF_6 will be evenly distributed throughout the entire house within 2.5 h and its concentration (C_0) in region 1 will be diluted to C_a with C_a/C_0 = 0.39 as shown in Fig. S3b. C_a is the average VOC concentration for the whole house, which can be mathematically determined as the exponential of the intercept of the fitted line in Fig. S3a. This dilution effect can explain why the concentration decay of SF_6 and CO do not follow a linear decay behavior (in log-scale y axis) in the initial 2.5 h after gas injections as seen in Fig. S3a and Fig. S3c. The mathematical solution of the dilution curve is solved in Eq. S1-S6.

Based on the mass balance in the house, C_a can be calculated by the equation below.

$$C_a = C_0 V/(V + V') \qquad \text{(Eq. S1)}$$

At any time t, the mass change in Region 1 can be represented as:

$$C_{t+\Delta t}V - C_t V = +C'_t Q\Delta t - C_t Q\Delta t, \qquad \text{(Eq. S2)}$$

and its derivation form is

$$\frac{dC}{dt} = \lambda_r(C' - C), \qquad \text{(Eq. S3)}$$

in which λ_r is the indoor recirculation rate (h^{-1}). Considering $C_0V = CV + C'V'$, Eq. S3 can be reformulated as:

$$\frac{dC}{dt} = -\lambda_r C(1 + V/V') + \lambda_r C_0\, V/V'. \qquad \text{(Eq. S4)}$$

To further simplify this equation, we assume $m = -\lambda_r(1 + V/V')$, $n = \lambda_r C_0\, V/V'$, $V_R = V/V'$, and therefore,

$$\frac{dC}{dt} = -mC + n, \qquad \text{(Eq. S5)}$$

and its mathematic solution is:

$$C = \frac{n - e^{-mt}(n-m)}{m} = \frac{V_R + e^{-\lambda_r(1+V_R)t}}{1+V_R} C_0, \qquad \text{(Eq. S6)}$$

Fig. S3b shows the dilution curve with a specific set of parameters of λ_r = 1.3 h^{-1} and V_R = 0.64. By incorporating this dilution process into the indoor-outdoor ventilation process as outlined in Eq. S7-S8, we were able to reproduce the observed decay of SF_6 and CO concentrations, as shown in Fig. S3c.

The modelled SF_6 decay is represented by equations below:

$$C_m = C_0 \cdot f_{di}(t) \cdot e^{-\lambda t}, \qquad \text{(Eq. S7)}$$

$$f_{di}(t) = \frac{V_R + e^{-\lambda_r(1+V_R)t}}{1+V_R}, \qquad \text{(Eq. S8)}$$

where C_m represents the modelled decay of SF_6 concentration, $f_{di}(t)$ represents a dilution function, and λ represents the ACR. This conceptual model highlights the potential influence of initial dilution on the observed concentration decay. Although the model assumes that indoor recirculation is the primary contributor to this dilution effect over hourly timescales, the actual indoor air mixing within the CASA house is intricate and includes factors such as natural gas diffusion, outdoor air infiltration, and indoor-outdoor ventilation. Thus, λ_r should be considered an effective air mixing rate (h^{-1}) that closely approximates the indoor recirculation rate of 1.3 h^{-1}.

Figure S4b displays the corrected SF_6 decay, which is solely driven by indoor-outdoor ventilation, while the observed decay is influenced by the dilution effect as well. Similarly, we corrected for the dilution effect on all smoke VOCs and CO measurements using Eq. S9. An example of the observed decay of C_6H_6O3 and its corrected decay is shown in Fig. S4b. When compared to the corrected decay, the original $C_6H_6O_3$ concentration decays more rapidly and is more diluted due to the dilution effect.

$$C_{obs} = C_{cor} \cdot f_{di}(t) \qquad \text{(Eq. S9)}$$

We note that this dilution effect caused by indoor air mixing should be considered carefully for the field/house studies in which we want to investigate gas-surface partitioning by injecting gases through single or multiple points. For example, when evaluating the ventilation item ($\Delta M = \lambda \int_0^t G(t)dt\, V$ in Eq. S11), $G(t)$ can be taken as either its lower limit of C' in Region 2 or its upper limit of C in Region 1, depending on the location of the venting air in the house. The deviation between C and C' is due to the initial VOC concentration gradient after smoke addition, which disappears as the room air becomes well-mixed by indoor mixing.

It is worth noting that while our dilution model is conceptual, the volume ratio of V/V' (= 0.64) derived for the smoke addition experiment closely matches, but is slightly higher than, the ratio (0.58) of the first-floor volume to the combined volume of the second-floor and basement. This finding is consistent with the observation that the smoke quickly mixes on the first floor, facilitated by the blowing fan, and a portion of it is also mixed to the second floor. Through modeling the concentrations of CO and SF_6, we determined that the gas mixing timescale for the first floor was approximately 6 minutes. Subsequently, the concentration decay would follow the SF_6 decay pattern, if assuming minimal or no surface partitioning occurred. Therefore, our model effectively captures the impact of these mixing processes on the observed concentration decays.

S3 A varying characteristic time (τ) of $C_6H_6O_3$ persistence

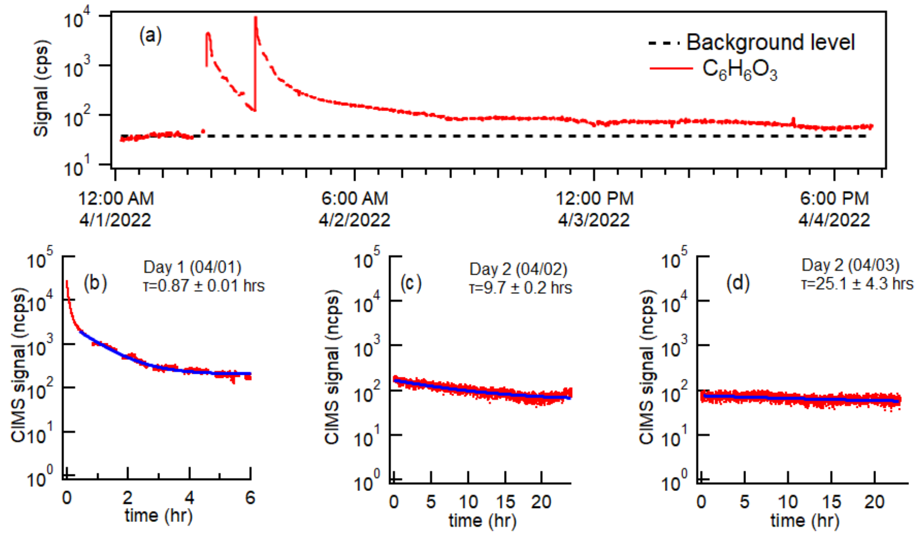

Fig. S5. (a) The concentration of a smoke tracer ($C_6H_6O_3$) following two smoke repeated injections remained elevated over four days. The dashed blacked line represents an average background level of $C_6H_6O_3$ before smoke additions began. (b), (c), and (d) show the fitted concentration decay and characteristic timescale (τ) on day 1, day 2 and day 3, respectively.

The changing time constant, τ, associated with the concentration decay reflects a complex and dynamic process for mass transfer of smoke VOCs in the indoor environment, including gas-phase processes such as gas diffusion, indoor recirculation, and ventilation, as well as multiphase processes (*52, 77, 78*) such as surface adsorption and desorption, surface-bulk mass transfer and bulk diffusion. Resolving these competing mechanisms is beyond the scope of this work. A practical challenge in determining τ in a field campaign is selecting an appropriate time period, as τ is constantly changing due to these coupled processes. A single τ value cannot accurately reflect to what extent the surface-gas partitioning occurs. For the initial hours when decay curves are steep, τ is affected by multiple processes and cannot be used to represent the partitioning process. Subsequently (> 2.5 h after smoke addition), decay curves flatten and τ values vary across VOCs as each VOC has different timescales for partitioning (i.e., different ADP values). In contrast, the ACP timescale reflects the overall partitioning behavior of smoke VOCs, with longer ACPs indicating larger K_{sa} and more mass distribution in surface sinks. ACPs demonstrate how each VOC interacts differently with indoor surfaces. ACPs are also not influenced by the initial dilution processes, as both the smoke VOCs and tracer gases (SF_6, CO) undergo the same initial processes, ensuring nearly identical ACPs regardless of whether the calculation is made before or after a dilution correction as seen in Fig. S4b.

S4 Examination of the chemical compositions of submicron aerosol and VOCs in fresh smoke additions.

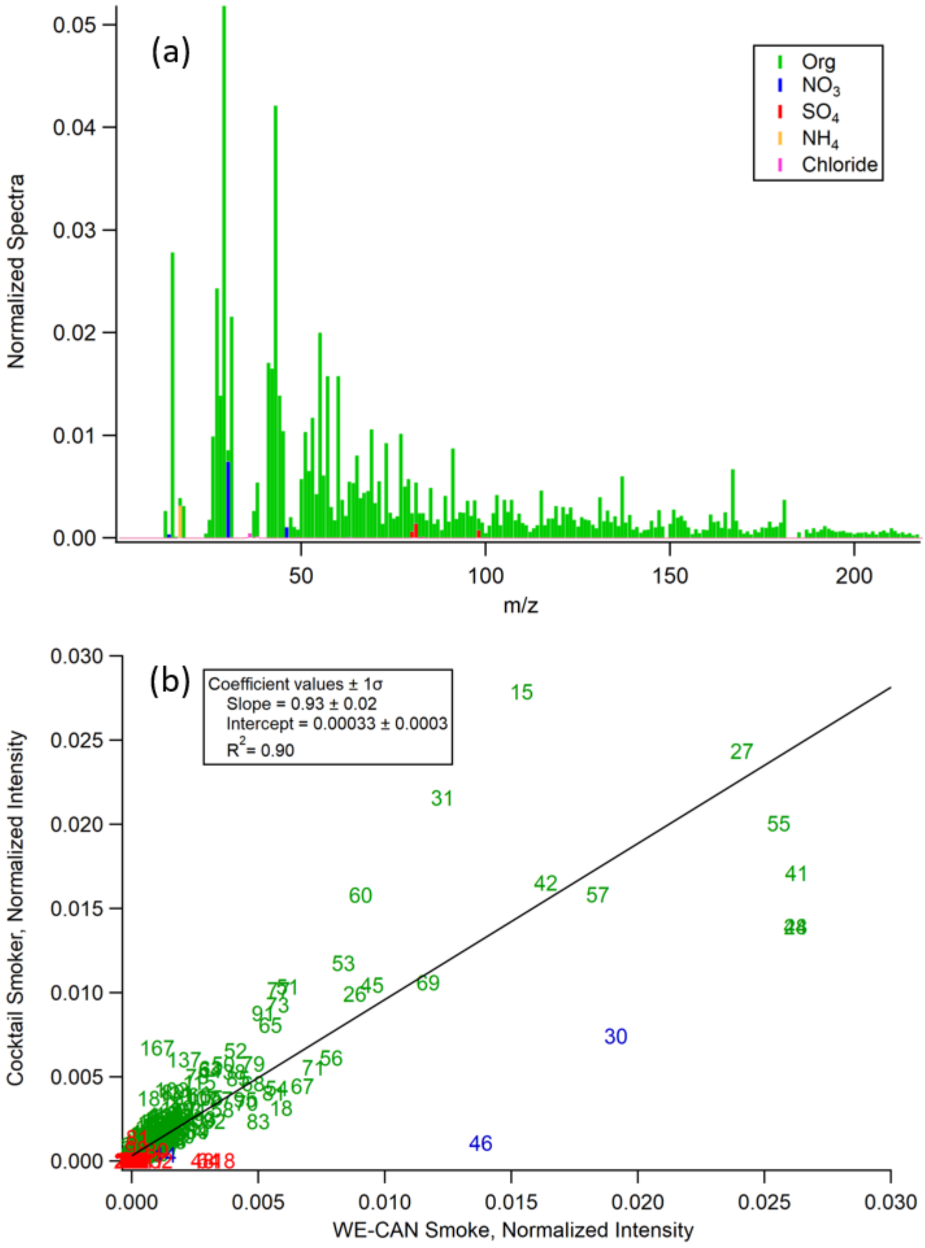

Fig.S6 From a bulk perspective, submicron organic aerosol of our CASA fresh smoke tends to be quite consistent with that of western wildfire emissions (*63*); particulate nitrate emissions are lower. (a) Normalized mass spectra of wood smoke generated by the cocktail smoker measured by the HR-ToF-AMS and summed to unit mass resolution. (b) Scatter plot of the normalized intensities of unit mass resolution (UMR) m/z ratios of wood smoke generated by the cocktail smoker versus real wildfire smoke measured during the WE-CAN flight campaign over the Western United States (R^2 = 0.901478).

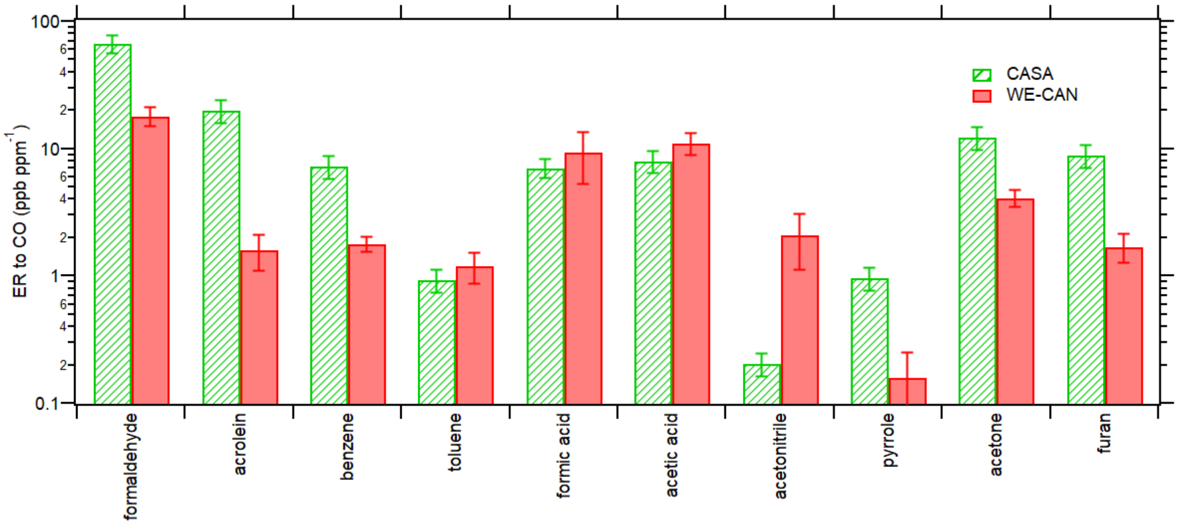

Fig.S7 Emission ratios (ERs) of a subset of VOCs measured in one of our smoke addition events. The error bars represent measurement uncertainties based on error propagation. These ratios are mostly consistent within one order of magnitude compared to those in WE-CAN project (*7*).

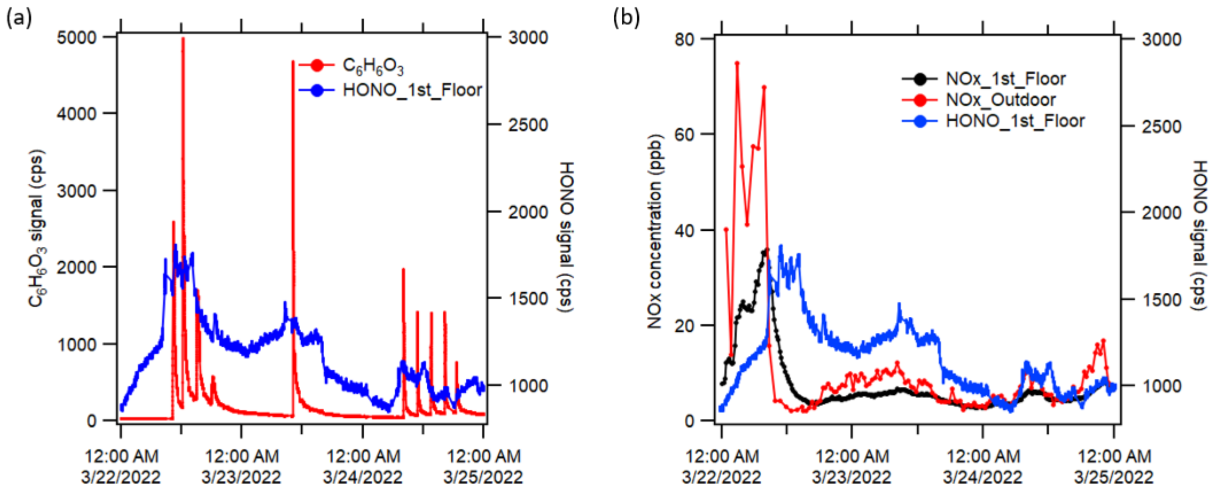

Fig. S8 (a) We observe no discernible correlation between HONO emissions and smoke addition events. $C_6H_6O_3$ is used as a tracer to illustrate the timing of these events. (b) The indoor HONO profile generally exhibits a similar trend to that observed in indoor and outdoor NO_x levels. The notable suppression of HONO and NOx generation when utilizing our cocktail smoker can be attributed to the comparatively lower temperatures achieved by smoldering wood chips in contrast to the flame temperature of natural gas in ambient air.

S5 The observed adsorption-desorption time scales on sampled indoor surfaces

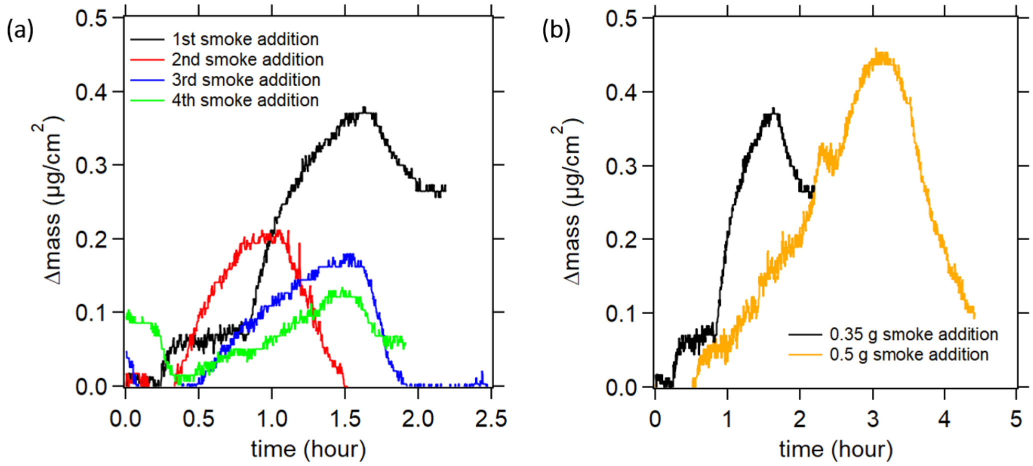

Fig. S9. The mass on a TiO_2 thin film measured by the quartz crystal microbalance changes after (a) each smoke addition. (b) the ADP timescales observed from the surface mass changes increase from 1.6 hours to 3.1 hours when more smoke is added into the house.

All five smoke additions depicted in Fig. S9 were direct smoke injections in which a cocktail smoker (Breville, BSM600SILUSC) generated smoke by burning pine woodchips. The four fresh smoke additions shown in Fig.S9(a) were conducted on 25 March 2022, at 165-minute intervals. Smoke additions occurred at 8:00 AM, 10:45 AM, 13:30 PM, and 16:15 PM local time. During each addition, smoke was rapidly injected into the first-floor hallway near the dining area of the house over a two-minute period while a fan was operated during the injection and for the following ten minutes to facilitate rapid mixing with the room air. In the dining area, a new TiO_2 thin film was positioned in the early morning to capture the effects of these four smoke additions. The mass of the surface film changed after each smoke addition in a consistent manner and with a similar time profile, consistent with the presence of dynamic gas-surface partitioning. To emphasize the observed partitioning behavior, the lowest mass value after each smoke addition was re-set to be zero, and the relative mass changes were showcased in the partitioning curves. Fig. 1D represents the average of the 1st, 3rd, and 4th smoke additions, with the exclusion of the 2nd addition due to a different smoke addition and mixing timeline, although this event exhibited a similar response to the other events.

Here, we describe the mathematical derivation of the correspondence of the ADP points between gas phase data and the surface mass data. For a specific VOC, the initial mass in the gas phase after smoke addition is M_0. As the indoor surface area is significantly larger (>1000x) than the particle surface area, and assuming the gas-particle partitioning process is not considered in this study, the VOC mass will be distributed into three mass sinks: the air ($G(t)V$), indoor surface reservoirs ($W(t)V$) and ventilation (ΔM), as outlined in the following equations.

$$M_0 = G(t)V + W(t)V + \Delta M \qquad \text{(Eq. S10)}$$

$$\Delta M = \lambda \int_0^t G(t)dt\, V \qquad \text{(Eq. S11)}$$

The gas phase data in our figures are normalized to the initial concentration G_0, and therefore

$$G_0 = \frac{M_0}{V} = G(t) + W(t) + \lambda \int_0^t G(t)dt \qquad \text{(Eq. S12)}$$

$$1 = \frac{G(t)}{G_0} + \frac{W(t)}{G_0} + \lambda \int_0^t \frac{G(t)}{G_0} dt \quad \text{(Eq. S13)}$$

$$1 = S_G(t) + S_W(t) + \lambda \int_0^t S_G(t) dt \quad \text{(Eq. S14)}$$

where $S_G(t)$ and $S_W(t)$ are normalized concentrations in gas sink and surface sink, respectively. At ADP equilibrium time t_e, S_W should reach its maximum value, and thus the derivative of the surface mass variation over time should be zero, as derived below:

$$S_W(t) = 1 - S_G(t) - \lambda \int_0^t S_G(t) dt \quad \text{(Eq. S15)}$$

$$\left.\frac{dS_W}{dt}\right|_{t=t_e} = \left.\frac{dS_G}{dt}\right|_{t=t_e} - \left.\frac{d\left(\lambda \int_0^t S_G(t)dt\right)}{dt}\right|_{t=t_e}$$

$$= \lambda S_G(t_e) - \lambda \left.\frac{\int_0^{t_e+dt} S_G(t)dt - \int_0^{t_e} S_G(t)dt}{dt}\right|_{t=t_e} = 0 \quad \text{(Eq. S16)}$$

Therefore, the time when S_W reaches its maximized value corresponds to the ADP time on the concentration decay curve.

In addition, the observed surface-air partitioning coefficient (K_{sa}) in this study is estimated from the experimental dataset by the equation below:

$$K_{sa} = \frac{W(t_e)\cdot(V/S)}{G(t_e)\cdot L} = \frac{S_W(t_e)}{S_G(t_e)} \cdot \frac{V}{S\cdot L} = \frac{1 - S_G(t_e) - \lambda \int_0^{t_e} S_G(t)dt}{S_G(t_e)} \cdot \frac{V}{S\cdot L} \quad \text{(Eq. S17)}$$

where *V/S* is the indoor volume-to-surface ratio, *L* is the indoor film thickness.

S6 Plotting ADP and ACP time scales with molecular properties

Table S1. Identified Compounds from Mass List of I-CIMS and PTR-MS

formula	molar masses (g/mol)	species	Log(C*)	ADP (h)	ACP (h)	*K*oa	OS_C
$C_2H_4IO_3^-$	76	peracetic acid(*8*)	8.00	1.1	6.7	3.7	1.00
$C_2H_6IO_3^-$	78	orthoacetic acid	6.23	2.6	8.4	5.4	0.00
$C_6H_6IO_2^-$	110	benzenediol(*7, 9*)	3.83	3.4	18.2	7.8	-0.33
$C_6H_6IO_3^-$	126	hydroxymethylfurfural(*7, 8*)	4.47	3.1	12.7	7.2	0.00
$C_6H_8IO_3^-$	128	furaneol	3.60	2.4	15.5	8.0	-0.33
$C_5H_8IO_4^-$	132	glutaric acid	3.37	3.0	14.7	8.3	0.00
$C_5H_8IO_3^-$	116	acetopropionic acid(*8*)	5.95	1.3	10.2	5.7	-0.40
$C_8H_8IO_2^-$	136	o-toluic acid(*7, 8*)	4.32	1.7	16.3	7.3	-0.50
$C_7H_8IO_2^-$	124	2-(hydroxymethyl)phenol(*8*)	3.44	5.3	21.2	8.2	-0.57
$C_4H_6IO_3^-$	102	acetoacetic acid(*8*)	6.01	1.0	9.2	5.6	0.00
$C_6H_8IO_4^-$	144	3-methylglutaconic acid	3.23	3.0	19.3	8.4	0.00
$C_7H_8IO_3^-$	140	3-methoxy-1,2-benzenediol	4.02	5.0	17.8	7.6	-0.29
$C_4H_5O^+$	68	furan(*7, 8, 49*)	9.34	2.5	7.2	2.3	-0.50
$C_4H_7O^+$	70	methyl vinyl ketone(*7, 8, 49*)	8.54	2.6	6.6	3.1	-1.00
$C_5H_5O^+$	81	cyclopentadienone(*7, 9*)	6.92	2.7	8.2	4.7	-0.40
$C_6H_9O^+$	96	2,5-dimethylfuran(*7, 8, 49*)	8.33	2.8	6.3	3.4	-1.00
$C_3H_5O^+$	56	acrolein(*7, 8, 49*)	8.90	2.6	4.8	2.8	-0.67
$C_4H_5O_2^+$	84	furanone(*7, 8, 49*)	6.65	2.5	10.7	5.0	0.00
$C_3H_7O_2^+$	74	hydroxyacetone(*7, 8*)	7.22	1.7	6.2	4.4	-0.67
$C_4H_7O_3^+$	102	acetoacetic acid(*8*)	6.01	3.1	12.0	5.6	0.00
$C_5H_9O_3^+$	116	acetopropionic acid(*8*)	5.95	3.7	13.6	5.7	-0.40
$C_8H_{11}O_2^+$	138	creosol(*7, 8*)	5.87	3.2	15.3	5.8	-0.75
$C_8H_{11}O^+$	122	2-ethylphenol,(*7*)	5.92	3.2	9.2	5.7	-1.00
$C_5H_9O^+$	84	3-methyl-3-buten-2-one(*7, 8*)	8.33	1.6	7.4	3.4	-1.20

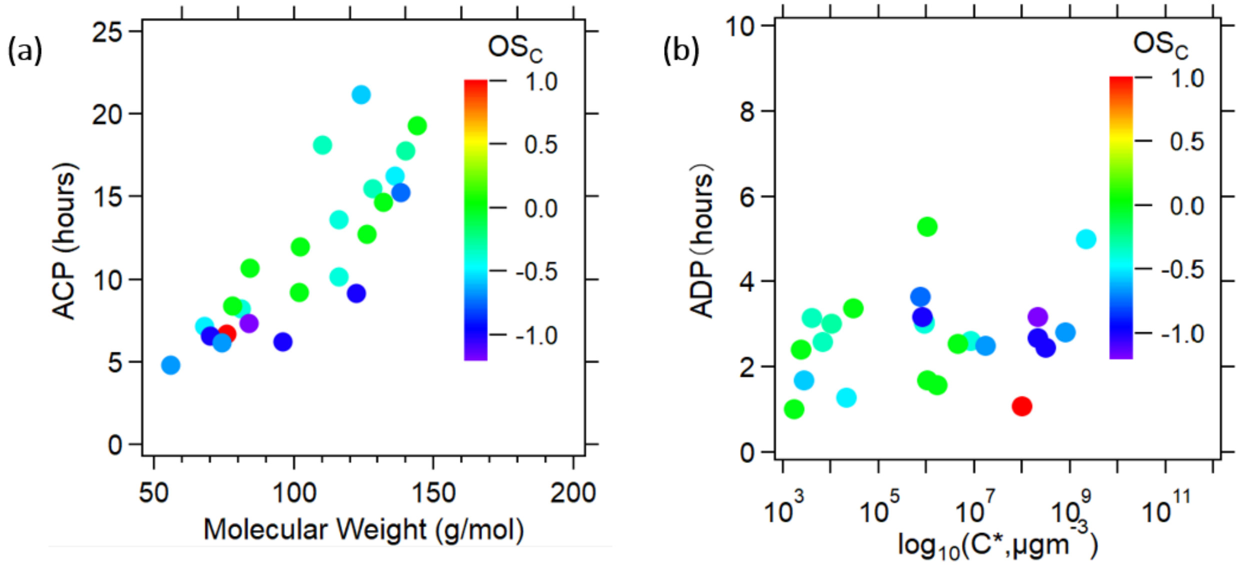

Fig. S10. (a) ACP time scales are plotted as a function of molecular weight for investigated VOCs. (b) ADP time scales are plotted as a function of saturation vapor pressure. The data points are colored by OS_C.

S7 Mass Distribution at ADP and ACP time for two ventilation scenarios

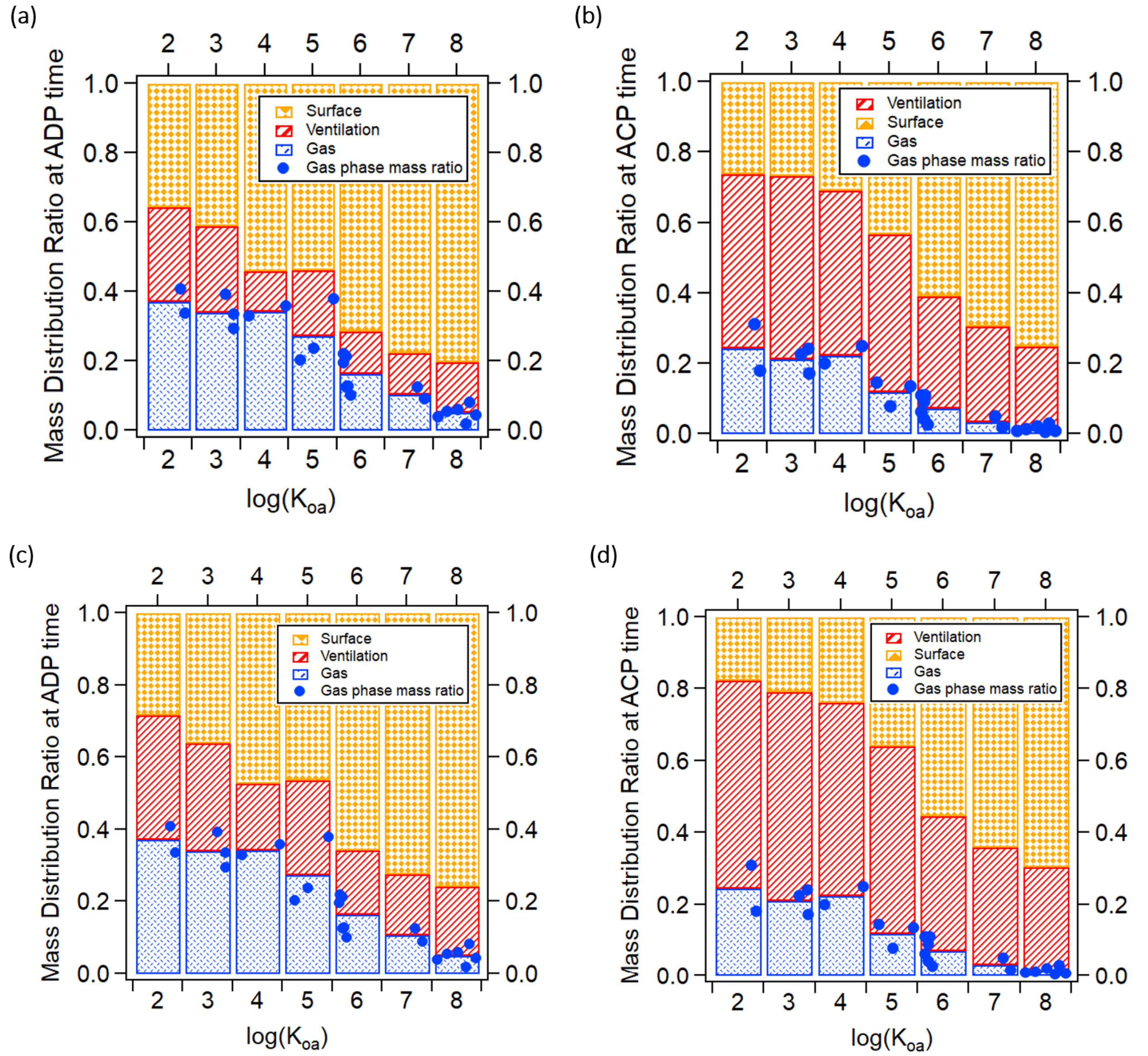

Fig. S11. Mass distributions across the three sinks of indoor air, surface reservoirs, and ventilation. The parameters for the average ventilation case are applied in (a) and (b). Parameters for the maximized ventilation case are applied in (c) and (d). The upper panels show mass distributions at the (a) ADP and (b) ACP timepoints for the average ventilation case. The lower panels show the mass distributions at (c) ADP and (d) ACP timepoints, respectively, for the maximized ventilation case. Note, for the average ventilation case, we assume that the VOC concentration in the ventilation term (Eq. S11) equals the average concentration of region 1 and region 2 in Fig. S4a; for the maximized ventilation case, we assume the VOC concentration in the ventilation term (Eq. S11) equals the concentration in region 1.

S8 The relationship between ACP time scales and the equilibrium mass/surface mass distribution

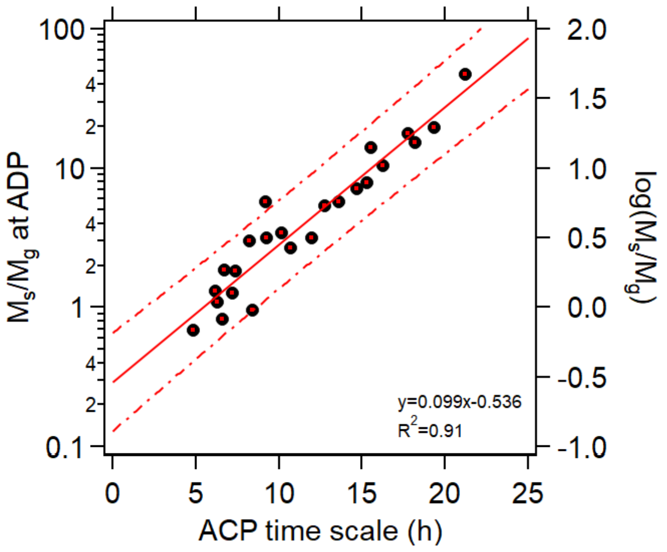

Fig. S12. The surface/gas mass ratio ($\log(M_s/M_g)$) correlates with the ACP time scales. An increase of 5 hours in ACP corresponds to a 3.1x increase in the surface/gas mass ratio. The ACP timescales are linearly correlated to ADP surface/mass ratio.

S9 Long-lasting impact of surface cleaning on indoor air quality

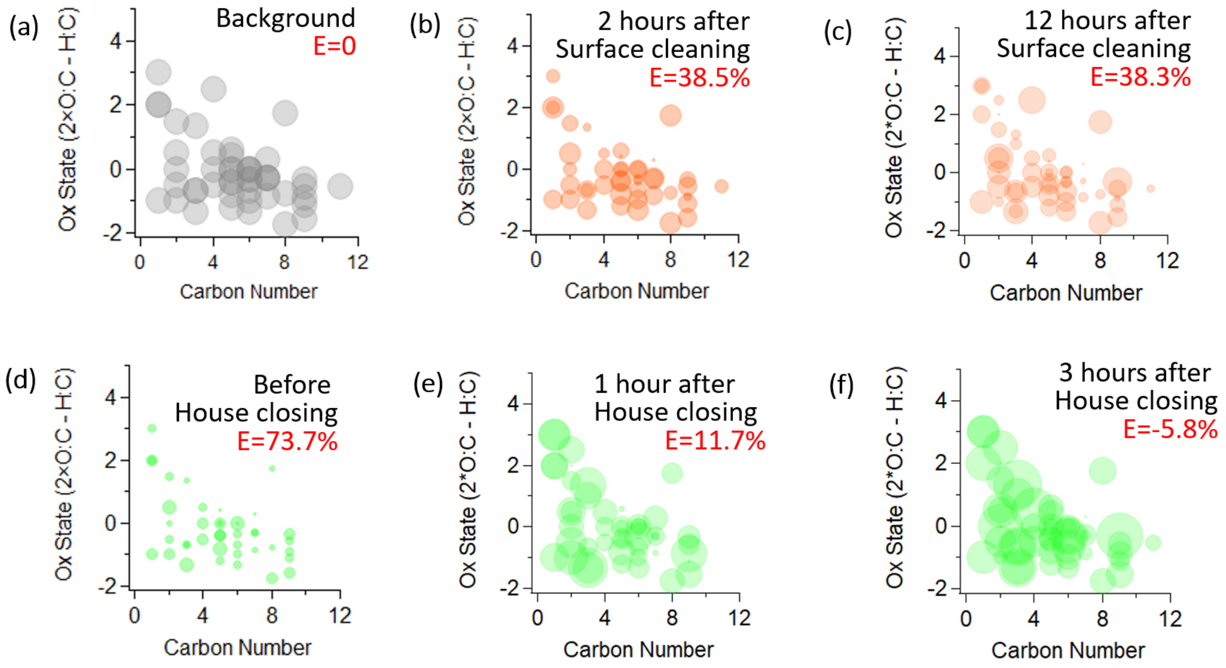

Fig. S13. Surface cleaning has a longer-lasting impact on air quality than window-opening. Panel (a) is a reference background case in which all circles are unity. Panels (b) and (c) illustrate the effectiveness of surface cleaning for CIMS-identified compounds at 2 hours and 12 hours after surface cleaning activities, respectively. Panels (d), (e), and (f) illustrate the effectiveness of window-opening for CIMS-identified chemicals at 0 hours, 1 hour, and 3 hours after closing all windows and the door, respectively.

S10 The effectiveness of house cleaning activities for the VOCUS-identified species

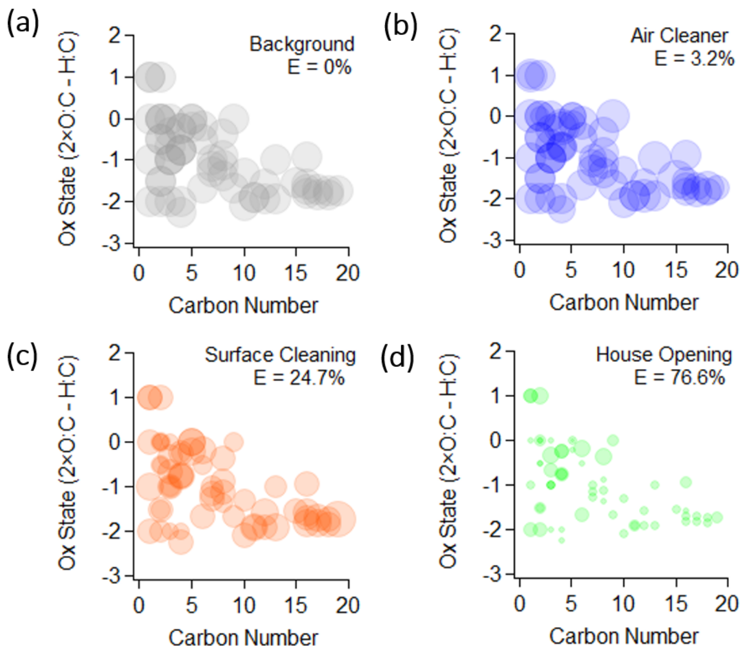

Fig. S14. The effectiveness of different house cleaning activities is shown for the VOCUS-identified species under (a) Background, (b) Running air cleaners, (c) Surface cleaning, and (d) Windows and door opening conditions.

S11 Surface cleaning is more effective for removing more oxidized species

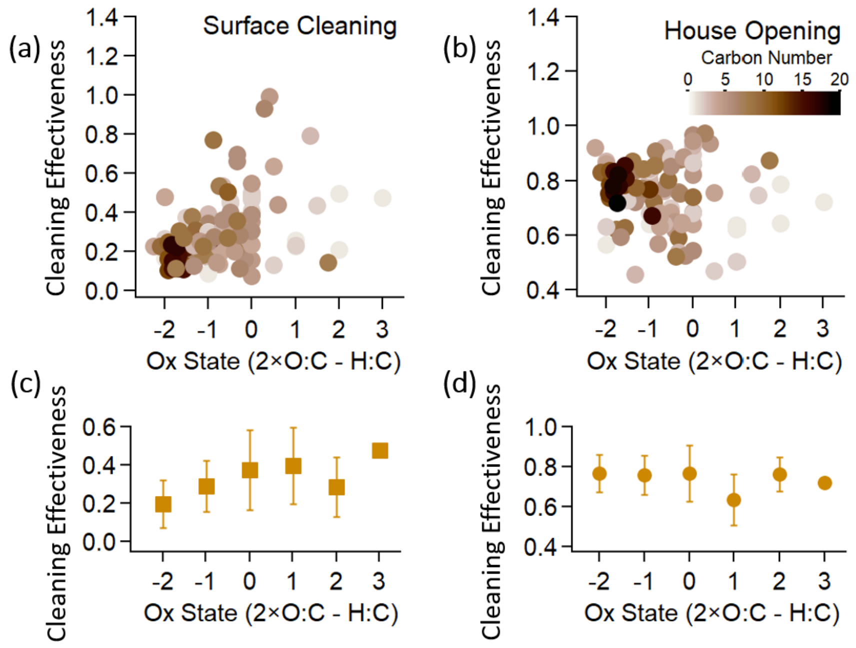

Fig. S15. We compare the cleaning effectiveness between the surface cleaning and the house opening over more than 100 chemicals identified from both CIMS and VOCUS measurement. (a) The cleaning effectiveness is plotted against the oxidation state (Ox State) for the surface cleaning case. The color of the data point represents the carbon number. (b) The cleaning effectiveness is plotted against the oxidation state (Ox State) for the house opening case. Panels (c) and (d) show the average effectiveness values binned by the Ox State for the surface cleaning case and house opening case, respectively.

S12 Estimation of VOC emission rates from surface reservoir and CADR of the applied air cleaner

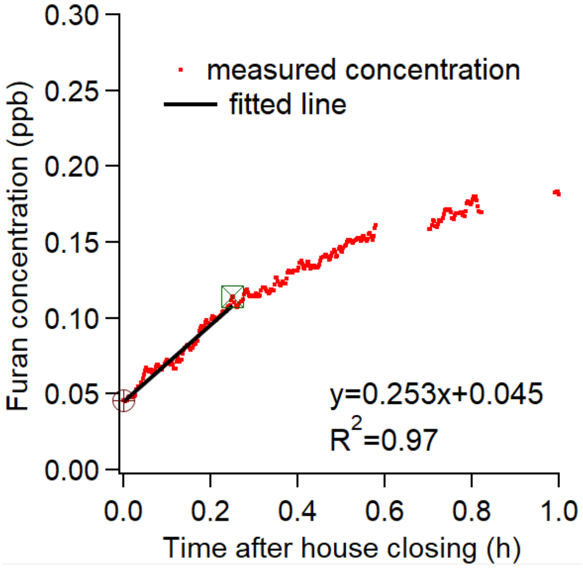

Fig. S16. Furan concentrations recover to pre-ventilation levels after closing the house. A linear fit to the trend between the indicated time points produces an estimate of furan emission rate from indoor surfaces.

Here we use furan as an example to compare the indoor furan emission rate with the removal rate of a commercial air cleaner. In Fig. 3A, indoor furan levels recover quickly after house closing at 4:30 pm due to furan emissions from indoor surfaces. Fig. S16 shows the magnified increase of furan concentration after house closing. Based on the initial 15 mins of data, we calculate an emission rate of 0.25 ppb h^{-1}, which equals 0.75 µg m^{-3} h^{-1}. Considering the volume of the house is 1484 m^3, the estimated furan emission rate for the whole house is 1113 µg h^{-1}. The furan concentration drops 4.8 % while running the plasma air cleaner, and we estimate the removal rate of the air cleaner is 53.4 µg h^{-1}. This estimation of removal rate represents an upper limit because the air cleaner is in the same room (i.e., kitchen) with the VOC measurement inlet and the removal effectiveness in other rooms may be lower than 4.8 % due to further distance from the air cleaner. We estimate a lower limit emission rate of 5.34 µg h^{-1} by multiplying the current removal rate with the volume ratio (~1/10) of the kitchen over the whole house, assuming no effect on other indoor space. The estimated CADR ranges from 7.8 m^3 h^{-1} to 78 m^3 h^{-1} based on the equation below:

$$\mathrm{CADR} = F \times E \quad \text{(Eq.S18)}$$

$$E = \frac{m_R}{F \times C_f} \quad \text{(Eq.S19)}$$

in which F is the flow rate of the air cleaner (276.9 m^3 h^{-1}), and E is the furan removal efficiency. E can be estimated by Eq. S19, in which C_f is the furan concentration before running air cleaners (0.68 µg m^{-3}), and m_R is the furan removal rate of the air cleaner, ranging from 5.34 µg h^{-1} to 53.4 µg h^{-1}. The largest source of uncertainty in this evaluation is the concentration gradient of furan in the house. Multiple measurement points will be useful in future studies to evaluate the performance of air cleaners more accurately in a single-family home. The derived CADR from this study is consistent with previous lab studies (*38*), in which the CADR of the consumer-grade air cleaners for VOCs (e.g., toluene and limonene) was determined to range from 0 m^3 h^{-1} to 45.8 m^3 h^{-1}.

S13 Technical specifications of the applied air cleaners

To assess the efficacy of various air purifiers, four consumer-grade portable air cleaners were procured from different manufacturers with prices ranging between $500 and $1000. These air cleaners were chosen to cover a broad spectrum of physical and chemical cleaning technologies, including dual polarity ions, photocatalytic oxidation (PCO), UV, electrostatic charge, HEPA filter, and activated carbon filter. Additionally, two custom-built Corsi-Rosenthal boxes were tested, one with standard MERV-13 filters and the other with charcoal filters. All air cleaners were situated in the dining and kitchen area during operation. A detailed examination of these air cleaners and their performance will be presented in a separate manuscript. A summary of the manufacturer's reported information for the air cleaner utilized on April 7th is in Table S2.

Table S2. Technical specifications and operating parameters of the air cleaner used during the CASA campaign on April 7th.

Cleaning technologies	Filters + Dual polarity ions	VOC CADR (cfm)	unknown
HEPA filter	99.99 % HEPA	Manufacture suggested maximum room area (sqft)	3300
Deodorization filter	Activated charcoal	AHAM verified room size (sqft)	~700
Filter replacement recommendation	12 months	Ozone emissions (ppb)	1
Operating flow rate (cfm)	163	Energy consumption (watt)	85
Particle CADR (cfm)	405	Agencies Certified	Energy Star / AHAM / CARB / UL / Prop65

Note: we use default units from manufactures, and below we show unit conversions to SI standards.
1 m^3 s^{-1} = 2118.88 cfm, 1 m^2 = 10.76 sqft.

S14 Background O_3 concentration in smoke addition experiments

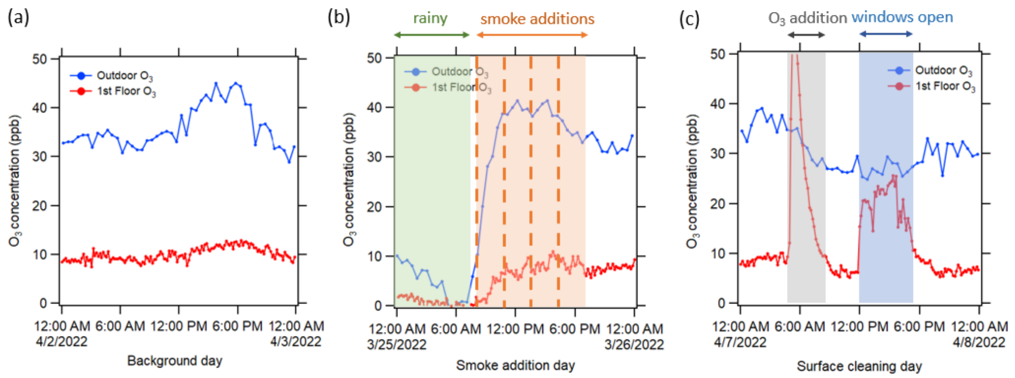

Fig. S17. O_3 concentrations on sample experimental days. (a) Background day. (b) Smoke addition day. Dashed lines represent smoke additions. (c) Surface cleaning day.

We present ozone levels in the house (and outdoor) below in Fig. S17. In thinking about whether indoor ozone levels impact measurements by making oxidized VOCs, we note that the levels of ozone are very stable throughout the experiment. This is due to the fact that in the absence of ozone additions, indoor ozone is driven by infiltration and follows outdoor ozone. These relatively stable and low (but representative of indoor residential environments) ozone concentrations mean that oxidation of volatile organic compounds should be low and part of the background. While ozone may be reacting with introduced smoke, the indoor levels are on the order of 10 ppb, which is low compared to the VOC concentrations, suggesting a minor effect on our observations. However, we did purposely select smoke VOCs that had stable backgrounds. Were ozone to be an order of magnitude greater, as in the case of ozone addition experiments, we would expect to see some additional oxidative loss of unsaturated VOCs combined with chemical production of oxidized VOCs.

REFERENCES AND NOTES

1. V. C. Radeloff, D. P. Helmers, H. A. Kramer, M. H. Mockrin, P. M. Alexandre, A. Bar-Massada, V. Butsic, T. J. Hawbaker, S. Martinuzzi, A. D. Syphard, S. I. Stewart, Rapid growth of the US wildland-urban interface raises wildfire risk. *Proc. Natl. Acad. Sci. U.S.A.* **115**, 3314–3319 (2018).

2. S. Hantson, N. Andela, M. L. Goulden, J. T. Randerson, Human-ignited fires result in more extreme fire behavior and ecosystem impacts. *Nat. Commun.* **13**, 2717 (2022).

3. National Academies of Sciences, Engineering, and Medicine, *The Chemistry of Fires at the Wildland-Urban Interface* (The National Academies Press, 2022), pp. 214.

4. R. Aguilera, T. Corringham, A. Gershunov, T. Benmarhnia, Wildfire smoke impacts respiratory health more than fine particles from other sources: Observational evidence from Southern California. *Nat. Commun.* **12**, 1493 (2021).

5. C. E. Reid, M. Brauer, F. H. Johnston, M. Jerrett, J. R. Balmes, C. T. Elliott, Critical review of health impacts of wildfire smoke exposure. *Environ. Health Perspect.* **124**, 1334–1343 (2016).

6. C. D. McClure, D. A. Jaffe, US particulate matter air quality improves except in wildfire-prone areas. *Proc. Natl. Acad. Sci. U.S.A.* **115**, 7901–7906 (2018).

7. W. Permar, Q. Wang, V. Selimovic, C. Wielgasz, R. J. Yokelson, R. S. Hornbrook, A. J. Hills, E. C. Apel, I.-T. Ku, Y. Zhou, B. C. Sive, A. P. Sullivan, J. L. Collett, T. L. Campos, B. B. Palm, Q. Y. Peng, J. A. Thornton, L. A. Garofalo, D. K. Farmer, S. M. Kreidenweis, E. J. T. Levin, P. J. DeMott, F. Flocke, E. V. Fischer, L. Hu, Emissions of trace organic gases from western U.S. wildfires based on WE-CAN aircraft measurements. *J. Geophys. Res. Atmos.* **126**, e2020JD033838 (2021).

8. S. J. Prichard, S. M. O'Neill, P. Eagle, A. G. Andreu, B. Drye, J. Dubowy, S. Urbanski, T. M. Strand, Wildland fire emission factors in North America: Synthesis of existing data, measurement needs and management applications. *Int. J. Wildland Fire* **29**, 132–147 (2020).

9. J. B. Gilman, B. M. Lerner, W. C. Kuster, P. D. Goldan, C. Warneke, P. R. Veres, J. M. Roberts, J. A. de Gouw, I. R. Burling, R. J. Yokelson, Biomass burning emissions and potential air quality impacts of

volatile organic compounds and other trace gases from fuels common in the US. *Atmos. Chem. Phys.* **15**, 13915–13938 (2015).

10. Y. T. Liang, D. Sengupta, M. J. Campmier, D. M. Lunderberg, J. S. Apte, A. H. Goldstein, Wildfire smoke impacts on indoor air quality assessed using crowdsourced data in California. *Proc. Natl. Acad. Sci. U.S.A.* **118**, e2106478118 (2021).

11. K. O'Dell, B. Ford, J. Burkhardt, S. Magzamen, S. C. Anenberg, J. Bayham, E. V. Fischer, J. R. Pierce, Outside in: The relationship between indoor and outdoor particulate air quality during wildfire smoke events in western US cities. *Environ. Res. Health* **1**, 015003 (2023).

12. K. P. Messier, L. G. Tidwell, C. C. Ghetu, D. Rohlman, R. P. Scott, L. M. Bramer, H. M. Dixon, K. M. Waters, K. A. Anderson, Indoor versus outdoor air quality during wildfires. *Environ. Sci. Technol. Lett.* **6**, 696–701 (2019).

13. G. Davison, K. K. Barkjohn, G. S. W. Hagler, A. L. Holder, S. Coefield, C. Noonan, B. Hassett-Sipple, Creating clean air spaces during wildland fire smoke episodes: Web summit summary. *Front. Public Health* **9**, 508971 (2021).

14. T. Javins, G. Robarge, E. G. Snyder, G. Nilsson, S. J. Emmerich, Protecting building occupants from smoke during wildfire and prescribed burn events. *ASHRAE J.* **63**, 38–43 (2021).

15. National Academies of Sciences, Engineering, and Medicine, *Why Indoor Chemistry Matters* (The National Academies Press, 2022), pp. 190.

16. J. P. D. Abbatt, G. C. Morrison, V. H. Grassian, M. Shiraiwa, C. J. Weschler, P. J. Ziemann, How should we define an indoor surface? *Indoor Air* **32**, e12955 (2022).

17. C. J. Weschler, W. W. Nazaroff, Semivolatile organic compounds in indoor environments. *Atmos. Environ.* **42**, 9018–9040 (2008).

18. J. P. D. Abbatt, C. Wang, The atmospheric chemistry of indoor environments. *Environ. Sci.: Processes Impacts* **22**, 25–48 (2020).

19. C. Wang, D. B. Collins, C. Arata, A. H. Goldstein, J. M. Mattila, D. K. Farmer, L. Ampollini, P. F. DeCarlo, A. Novoselac, M. E. Vance, W. W. Nazaroff, J. P. D. Abbatt, Surface reservoirs dominate dynamic gas-surface partitioning of many indoor air constituents. *Sci. Adv.* **6**, eaay8973 (2020).

20. D. M. Lunderberg, K. Kristensen, Y. Tian, C. Arata, P. K. Misztal, Y. Liu, N. Kreisberg, E. F. Katz, P. F. DeCarlo, S. Patel, M. E. Vance, W. W. Nazaroff, A. H. Goldstein, Surface emissions modulate indoor SVOC concentrations through volatility-dependent partitioning. *Environ. Sci. Technol.* **54**, 6751–6760 (2020).

21. M. Sleiman, J. M. Logue, W. T. Luo, J. F. Pankow, L. A. Gundel, H. Destaillats, Inhalable constituents of thirdhand tobacco smoke: Chemical characterization and health impact considerations. *Environ. Sci. Technol.* **48**, 13093–13101 (2014).

22. A. Manuja, J. Ritchie, K. Buch, Y. X. Wu, C. M. A. Eichler, J. C. Little, L. C. Marr, Total surface area in indoor environments. *Environ. Sci. Process. Impacts* **21**, 1384–1392 (2019).

23. P. S. J. Lakey, C. M. A. Eichler, C. Y. Wang, J. C. Little, M. Shiraiwa, Kinetic multi-layer model of film formation, growth, and chemistry (KM-FILM): Boundary layer processes, multi-layer adsorption, bulk diffusion, and heterogeneous reactions. *Indoor Air* **31**, 2070–2083 (2021).

24. Y. Liu, A. G. Bé, V. W. Or, M. R. Alves, V. H. Grassian, F. M. Geiger, Challenges and opportunities in molecular-level indoor surface chemistry and physics. *Cell Rep. Phys. Sci.* **1**, 100256 (2020).

25. C. Y. Lim, J. P. Abbatt, Chemical composition, spatial homogeneity, and growth of indoor surface films. *Environ. Sci. Technol.* **54**, 14372–14379 (2020).

26. L. B. Algrim, D. Pagonis, J. A. de Gouw, J. L. Jimenez, P. J. Ziemann, Measurements and modeling of absorptive partitioning of volatile organic compounds to painted surfaces. *Indoor Air* **30**, 745–756 (2020).

27. B. L. Deming, P. J. Ziemann, Quantification of alkenes on indoor surfaces and implications for chemical sources and sinks. *Indoor Air* **30**, 914–924 (2020).

28. A. P. Ault, V. H. Grassian, N. Carslaw, D. B. Collins, H. Destaillats, D. J. Donaldson, D. K. Farmer, J. L. Jimenez, V. F. McNeill, G. C. Morrison, R. E. O'Brien, M. Shiraiwa, M. E. Vance, J. R. Wells, W. Xiong, Indoor surface chemistry: Developing a molecular picture of reactions on indoor interfaces. *Chem* **6**, 3203–3218 (2020).

29. C. J. Weschler, W. W. Nazaroff, Growth of organic films on indoor surfaces. *Indoor Air* **27**, 1101–1112 (2017).

30. R. B. Jorgensen, O. Bjorseth, B. Malvik, Chamber testing of adsorption of volatile organic compounds (VOCs) on material surfaces. *Indoor Air* **9**, 2–9 (1999).

31. D. Won, R. L. Corsi, M. Rynes, Sorptive interactions between VOCs and indoor materials. *Indoor Air* **11**, 246–256 (2001).

32. B. A. Tichenor, Z. Guo, J. E. Dunn, L. E. Sparks, M. A. Mason, The Interaction of vapour phase organic compounds with indoor sinks. *Indoor Air* **1**, 23–35 (1991).

33. F. Thevenet, M. Verriele, P. Harb, S. Thlaijeh, R. Brun, M. Nicolas, S. Angulo-Milhem, The indoor fate of terpenes: Quantification of the limonene uptake by materials. *Build. Environ.* **188**, 107433 (2021).

34. M. Rizk, M. Verriele, S. Dusanter, C. Schoemaecker, S. Le Calve, N. Locoge, Fast sorption measurements of volatile organic compounds on building materials: Part 1-Methodology developed for field applications. *Build. Environ.* **99**, 200–209 (2016).

35. B. C. Singer, A. T. Hodgson, T. Hotchi, K. Y. Ming, R. G. Sextro, E. E. Wood, N. J. Brown, Sorption of organic gases in residential rooms. *Atmos. Environ.* **41**, 3251–3265 (2007).

36. B. C. Singer, K. L. Revzan, T. Hotchi, A. T. Hodgson, N. J. Brown, Sorption of organic gases in a furnished room. *Atmos. Environ.* **38**, 2483–2494 (2004).

37. D. B. Collins, R. F. Hems, S. M. Zhou, C. Wang, E. Grignon, M. Alavy, J. A. Siegel, J. P. D. Abbatt, Evidence for gas-surface equilibrium control of indoor nitrous acid. *Environ. Sci. Technol.* **52**, 12419–12427 (2018).

38. Q. Ye, J. E. Krechmer, J. D. Shutter, V. P. Barber, Y. Li, E. Helstrom, L. J. Franco, J. L. Cox, A. I. H. Hrdina, M. B. Goss, N. Tahsini, M. Canagaratna, F. N. Keutsch, J. H. Kroll, Real-time laboratory measurements of VOC emissions, removal rates, and byproduct formation from consumer-grade oxidation-based air cleaners. *Environ. Sci. Technol. Lett.* **8**, 1020–1025 (2021).

39. D. B. Collins, D. K. Farmer, Unintended consequences of air cleaning chemistry. *Environ. Sci. Technol.* **55**, 12172–12179 (2021).

40. J. M. Mattila, P. S. J. Lakey, M. Shiraiwa, C. Wang, J. P. D. Abbatt, C. Arata, A. H. Goldstein, L. Ampollini, E. F. Katz, P. F. DeCarlo, S. Zhou, T. F. Kahan, F. J. Cardoso-Saldana, L. H. Ruiz, A. Abeleira, E. K. Boedicker, M. E. Vance, D. K. Farmer, Multiphase chemistry controls inorganic chlorinated and nitrogenated compounds in indoor air during bleach cleaning. *Environ. Sci. Technol.* **54**, 1730–1739 (2020).

41. D. Poppendieck, M. Y. Gong, S. Zimmerman, L. Ng, Evaluation of a four-zone indoor exposure model for predicting TCPP concentrations in a low-energy test house. *Build. Environ.* **199**, 107888 (2021).

42. A. H. Fanney, W. Healy, V. Payne, J. Kneifel, L. Ng, B. Doughety, T. Ullah, F. Omar, Small changes yield large results at NIST's net-zero energy residential test facility. *J. Sol. Energy Eng. Trans. ASME* **139**, 061009 (2017).

43. R. Habre, D. C. Dorman, J. Abbatt, W. P. Bahnfleth, E. Carter, D. Farmer, G. Gawne-Mittelstaedt, A. H. Goldstein, V. H. Grassian, G. Morrison, J. Peccia, D. Poppendieck, K. A. Prather, M. Shiraiwa, H. M. Stapleton, M. Williams, M. E. Harries, Why indoor chemistry matters: A national academies consensus report. *Environ. Sci. Technol.* **56**, 10560–10563 (2022).

44. H. Schwartz-Narbonne, D. J. Donaldson, Water uptake by indoor surface films. *Sci. Rep.* **9**, 11089 (2019).

45. G. Rubasinghege, V. H. Grassian, Role(s) of adsorbed water in the surface chemistry of environmental interfaces. *Chem. Commun.* **49**, 3071–3094 (2013).

46. W. J. Wei, C. Mandin, O. Ramalho, Influence of indoor environmental factors on mass transfer parameters and concentrations of semi-volatile organic compounds. *Chemosphere* **195**, 223–235 (2018).

47. K. Kristensen, D. M. Lunderberg, Y. J. Liu, P. K. Misztal, Y. L. Tian, C. L. Arata, W. W. Nazaroff, A. H. Goldstein, Sources and dynamics of semivolatile organic compounds in a single-family residence in northern California. *Indoor Air* **29**, 645–655 (2019).

48. N. M. Donahue, J. H. Kroll, S. N. Pandis, A. L. Robinson, A two-dimensional volatility basis set - Part 2: Diagnostics of organic-aerosol evolution. *Atmos. Chem. Phys.* **12**, 615–634 (2012).

49. B. Yuan, A. R. Koss, C. Warneke, M. Coggon, K. Sekimoto, J. A. de Gouw, Proton-transfer-reaction mass spectrometry: Applications in atmospheric sciences. *Chem. Rev.* **117**, 13187–13229 (2017).

50. H. Xiao, F. Wania, Is vapor pressure or the octanol-air partition coefficient a better descriptor of the partitioning between gas phase and organic matter? *Atmos. Environ.* **37**, 2867–2878 (2003).

51. R. Meininghaus, E. Uhde, Diffusion studies of VOC mixtures in a building material. *Indoor Air* **12**, 215–222 (2002).

52. M. Shiraiwa, U. Poschl, Mass accommodation and gas-particle partitioning in secondary organic aerosols: Dependence on diffusivity, volatility, particle-phase reactions, and penetration depth. *Atmos. Chem. Phys.* **21**, 1565–1580 (2021).

53. C. Arata, P. K. Misztal, Y. L. Tian, D. M. Lunderberg, K. Kristensen, A. Novoselac, M. E. Vance, D. K. Farmer, W. W. Nazaroff, A. H. Goldstein, Volatile organic compound emissions during HOMEChem. *Indoor Air* **31**, 2099–2117 (2021).

54. California Energy Commission, *Buiding Energy Efficiency Standards for Residential and Nonresidential Buildings* (California Energy Commission, 2018).

55. S. M. Duncan, S. Tomaz, G. Morrison, M. Webb, J. Atkin, J. D. Surratt, B. J. Turpin, Dynamics of residential water-soluble organic gases: Insights into sources and sinks. *Environ. Sci. Technol.* **53**, 1812–1821 (2019).

56. H. Schwartz-Narbonne, J. P. D. Abbatt, P. F. DeCarlo, D. K. Farmer, J. M. Mattila, C. Wang, D. J. Donaldson, J. A. Siegel, Modeling the removal of water-soluble trace gases from indoor air via air conditioner condensate. *Environ. Sci. Technol.* **55**, 10987–10993 (2021).

57. A. L. Hodshire, E. Carter, J. M. Mattila, V. Ilacqua, J. Zambrana, J. P. D. Abbatt, A. Abeleira, C. Arata, P. F. DeCarlo, A. H. Goldstein, L. H. Ruiz, M. E. Vance, C. Wang, D. K. Farmer, Detailed investigation of the contribution of gas-phase air contaminants to exposure risk during indoor activities. *Environ. Sci. Technol.* **56**, 12148–12157 (2022).

58. J. M. Mattila, C. Arata, A. Abeleira, Y. Zhou, C. Wang, E. F. Katz, A. H. Goldstein, J. P. D. Abbatt, P. F. DeCarlo, M. E. Vance, D. K. Farmer, Contrasting chemical complexity and the reactive organic carbon budget of indoor and outdoor air. *Environ. Sci. Technol.* **56**, 109–118 (2021).

59. D. J. Price, D. A. Day, D. Pagonis, H. Stark, L. B. Algrim, A. V. Handschy, S. Liu, J. E. Krechmer, S. L. Miller, J. F. Hunter, J. A. de Gouw, P. J. Ziemann, J. L. Jimenez, Budgets of organic carbon composition and oxidation in indoor air. *Environ. Sci. Technol.* **53**, 13053–13063 (2019).

60. B. Stephens, E. T. Gall, M. Heidarinejad, D. K. Farmer, Interpreting air cleaner performance data. *ASHRAE J.* **64**, 20–30 (2022).

61. C. Wang, B. Bottorff, E. Reidy, C. M. F. Rosales, D. B. Collins, A. Novoselac, D. K. Farmer, M. E. Vance, P. S. Stevens, J. P. D. Abbatt, Cooking, bleach cleaning, and air conditioning strongly impact levels of HONO in a house. *Environ. Sci. Technol.* **54**, 13488–13497 (2020).

62. A. Moravek, T. C. VandenBoer, Z. Finewax, D. Pagonis, B. A. Nault, W. L. Brown, D. A. Day, A. V. Handschy, H. Stark, P. Ziemann, J. L. Jimenez, J. A. de Gouw, C. J. Young, Reactive chlorine emissions from cleaning and reactive nitrogen chemistry in an indoor athletic facility. *Environ. Sci. Technol.* **56**, 15408–15416 (2022).

63. L. A. Garofalo, M. A. Pothier, E. J. T. Levin, T. Campos, S. M. Kreidenweis, D. K. Farmer, Emission and evolution of submicron organic aerosol in smoke from wildfires in the Western United States. *ACS Earth Space Chem.* **3**, 1237–1247 (2019).

64. American National Red Cross, *Cleaning Up After a Fire.* (American National Red Cross, 2022); www.redcross.org/get-help/how-to-prepare-for-emergencies/types-of-emergencies/fire/cleaning-up-after-fire.html.

65. B. H. Lee, F. D. Lopez-Hilfiker, C. Mohr, T. Kurtén, D. R. Worsnop, J. A. Thornton, An iodide-adduct high-resolution time-of-flight chemical-ionization mass spectrometer: Application to atmospheric inorganic and organic compounds. *Environ. Sci. Technol.* **48**, 6309–6317 (2014).

66. P. Brophy, D. K. Farmer, A switchable reagent ion high resolution time-of-flight chemical ionization mass spectrometer for real-time measurement of gas phase oxidized species: characterization from the 2013 southern oxidant and aerosol study. *Atmos. Meas. Tech.* **8**, 2945–2959 (2015).

67. S. Tomaz, T. Q. Cui, Y. Z. Chen, K. G. Sexton, J. M. Roberts, C. Warneke, R. J. Yokelson, J. D. Surratt, B. J. Turpin, Photochemical cloud processing of primary wildfire emissions as a potential source of secondary organic aerosol. *Environ. Sci. Technol.* **52**, 11027–11037 (2018).

68. S. M. Duncan, K. G. Sexton, B. J. Turpin, Oxygenated VOCs, aqueous chemistry, and potential impacts on residential indoor air composition. *Indoor Air* **28**, 198–212 (2018).

69. C. J. Hennigan, M. H. Bergin, A. G. Russell, A. Nenes, R. J. Weber, Gas/particle partitioning of water-soluble organic aerosol in Atlanta. *Atmos. Chem. Phys.* **9**, 3613–3628 (2009).

70. J. Schuttlefield, H. Al-Hosney, A. Zachariah, V. H. Grassian, Attenuated total reflection Fourier transform infrared spectroscopy to investigate water uptake and phase transitions in atmospherically relevant particles. *Appl. Spectrosc.* **61**, 283–292 (2007).

71. J. D. Schuttlefield, D. Cox, V. H. Grassian, An investigation of water uptake on clays minerals using ATR-FTIR spectroscopy coupled with quartz crystal microbalance measurements. *J. Geophys. Res-Atmos.* **112**, D21303 (2007).

72. A. H. Fanney, V. Payne, T. Ullah, L. Ng, M. Boyd, F. Omar, M. Davis, H. Skye, B. Dougherty, B. Polidoro, W. Healy, J. Kneifel, B. Pettit, Net-zero and beyond! Design and performance of NIST's net-zero energy residential test facility. *Energ. Buildings* **101**, 95–109 (2015).

73. L. C. Ng, S. Zimmerman, J. Good, B. Toll, S. J. Emmerich, A. K. Persily, Estimating real-time infiltration for use in residential ventilation control. *Indoor Built Environ.* **29**, 508–526 (2020).

74. P. Brophy, D. K. Farmer, Clustering, methodology, and mechanistic insights into acetate chemical ionization using high-resolution time-of-flight mass spectrometry. *Atmos. Meas. Tech.* **9**, 3969–3986 (2016).

75. F. D. Lopez-Hilfiker, S. Iyer, C. Mohr, B. H. Lee, E. L. D'Ambro, T. Kurten, J. A. Thornton, Constraining the sensitivity of iodide adduct chemical ionization mass spectrometry to multifunctional organic molecules using the collision limit and thermodynamic stability of iodide ion adducts. *Atmos. Meas. Tech.* **9**, 1505–1512 (2016).

76. J. E. Krechmer, D. Pagonis, P. J. Ziemann, J. L. Jimenez, Quantification of gas-wall partitioning in teflon environmental chambers using rapid bursts of low-volatility oxidized species generated in situ. *Environ. Sci. Technol.* **50**, 5757–5765 (2016).

77. U. Pöschl, M. Shiraiwa, Multiphase chemistry at the atmosphere-biosphere interface influencing climate and public health in the anthropocene. *Chem. Rev.* **115**, 4440–4475 (2015).

78. U. Pöschl, Y. Rudich, M. Ammann, Kinetic model framework for aerosol and cloud surface chemistry and gas-particle interactions–Part 1: General equations, parameters, and terminology. *Atmos. Chem. Phys.* **7**, 5989–6023 (2007).
